# Prioritising opportunities to strengthen the maternal, newborn, and child health research ecosystem in Ethiopia: a Delphi exercise

**DOI:** 10.7189/jogh.16.04001

**Published:** 2026-01-23

**Authors:** Lisanu Taddesse, Michelle L Korte, Bezawit Mesfin Hunegnaw, Habtamu Teklie, Delayehu Bekele, Getachew Tolera, Meseret Zelalem, Grace J Chan

**Affiliations:** 1 HaSET Maternal and Child Health Research Program, Addis Ababa, Ethiopia; 2 Department of Global Health and Population, Harvard T.H. Chan School of Public Health, Boston, USA; 3 Ethiopia Public Health Institute, Addis Ababa, Ethiopia; 4 Department of Obstetrics and Gynecology, St. Paul’s Hospital Millennium Medical College, Addis Ababa, Ethiopia; 5 The African Centre for Early Childhood Development, Addis Ababa, Ethiopia; 6 Department of Pediatrics, Children’s Hospital of Philadelphia, Perelman School of Medicine, University of Pennsylvania, Philadelphia, Pennsylvania, USA

## Abstract

**Background:**

Quality research is essential to improving maternal, newborn, and child health (MNCH). Although Ethiopia has rapidly expanded academic and research institutions, duplication of studies, gaps in prioritisation and methods, and limited guidance on utilising evidence inhibit a coordinated approach to informing MNCH policy. We aim to address these challenges by characterising and prioritising the needs and opportunities of the MNCH research ecosystem in Ethiopia.

**Methods:**

We purposively sampled experts for a three-stage Delphi study. Key informant interviews (KIIs) (n = 18) explored needs and challenges in capacity-strengthening, community engagement in research, operational infrastructure, collaborations, and funding. We thematically coded KII responses to generate 134 statements, which were then rated in an anonymous questionnaire (n = 34) on a Likert scale. We calculated average scores and percentage agreement for each statement. Finally, consensus-building discussions (n = 28) identified top priorities within each topic.

**Results:**

Average percentage agreement across statements was 87% (range = 37–100). Highly endorsed priorities included strengthening inclusivity in research agenda-setting, prioritising research addressing key MNCH needs, enhancing research training by emphasising local experiences, cultivating intellectual curiosity, building skills in data analysis and translation, fostering research collaborations with greater multidisciplinary expertise, long-term mentorship, and capacity-building for local institutions, and engaging communities more effectively.

**Conclusions:**

Understanding challenges in the existing research environment will enable better-informed activities and stronger research networks that address local priorities. We characterised the MNCH research ecosystem across multiple dimensions, offering actionable opportunities to strengthen research capacities, infrastructure, and innovation design and evaluation through advocacy, organisational and system strengthening efforts, curricula development, and the implementation of principles to guide partnerships and agenda-setting for a variety of stakeholders. Future efforts should prioritise fostering a culture of evidence, collaborative prioritisation of research between policymakers and researchers, and sustained commitment to scaling evidence-based practices to advance MNCH outcomes.

Ethiopia is one of six countries accounting for more than half of global under-five deaths, with 178 000 child deaths in 2022 – 107 000 of which occur in the neonatal period [1]. Additionally, 10 000 women died in 2020 from mostly preventable causes related to pregnancy and childbirth [1]. Improving such a health situation requires policies that are well-targeted and well-enacted, which in turn rely on a strong body of research and evidence that is locally tailored and appropriate. Strengthening the capacity of researchers – and the broader ecosystem that enables their development and contributions – is thus foundational to improving health in countries like Ethiopia, which bear a significant burden of morbidity and mortality.

Over the past few decades, Ethiopia has rapidly expanded its number of academic and research institutions while emphasising its commitment to maternal, newborn, and child health (MNCH). With this growth has come a rapid increase in MNCH research, with 70% of the 2170 studies published between 1946 and 2018 published in the last decade alone [2]. The quality of MNCH research, however, varies across and within institutions, and translating research into policies and programming remains limited. Ethiopia’s Health Sector Transformation Plan II concludes that most findings from research studies are not used in policy and decision-making, highlighting a lack of coordination between universities' research priorities and the policy directions of the Ministry of Health (MoH) [3]. Research prioritisation does not take place regularly or transparently, and research activities tend to be mostly descriptive rather than operational. Researchers are not sufficiently supported throughout the research-to-policy and practice lifecycle, and institutional incentives and mechanisms to promote research from the ground up by regional health bureaus, zonal health departments, hospitals, and lower-level health institutions are lacking.

While the importance of strengthening research capacity as an input to improved health outcomes is well acknowledged, several barriers hinder effective capacity development. Overall, the MNCH research environment is constrained by both poor coordination and limited resources, with a reliance on donor funding that may not be responsive to local priorities [4] and minimal domestic expenditure on health research; as of 2017, Ethiopia spent only 0.27% of its gross domestic product on research and development across sectors [5]. Moreover, the evidence base for health research capacity strengthening (RCS) is limited [6], and while numerous health RCS initiatives, both local and international, have taken place in Ethiopia over the years, there has been no assessment of the key needs and priorities within the MNCH research environment.

To address this gap, the MoH, in collaboration with the HaSET (‘happiness’ in Amharic) Maternal and Child Health Research Program, conducted a formative assessment to identify priorities for strengthening the MNCH research ecosystem. HaSET is a collaborative effort across St. Paul's Hospital Millennium Medical College, the Ethiopian Public Health Institute (EPHI), and Harvard T.H. Chan School of Public Health, established to improve MNCH evidence generation and its translation into policy and practice in Ethiopia [7]. One objective of the formative assessment was to identify specific priorities for MNCH research in Ethiopia, which are described elsewhere [8]. We characterise the MNCH research ecosystem in Ethiopia and identify opportunities for stakeholders to strengthen it toward the sustainable production of high-quality, impactful research. While definitions vary, we use the term ‘capacity-strengthening’ in this paper to refer to efforts to strengthen individual, institutional, and ecosystem capacities to produce and utilise high-quality research in both the short and long-term to achieve transformational empowerment and sustainability; this definition is informed by those set out by the World Health Organization [9] and described in a scoping review of health RCS [6]. Specifically, our assessment of the MNCH research ecosystem spans research capacity development and training, agenda-setting and research prioritisation, networks and partnerships, community engagement, operational infrastructure, and funding opportunities.

## METHODS

We conducted this study from April 2020 to February 2021, leveraging an adapted Delphi approach comprising a round of key informant interviews (KIIs), an online questionnaire, and consensus-building discussions to identify key challenges in the MNCH research environment in Ethiopia and prioritise capacity-strengthening needs and opportunities.

The Delphi method is an iterative, multi-stage process used to transform individual opinions into group consensus [10]. Typically, an initial questionnaire or interview collects qualitative comments that are fed back to participants through a quantitative questionnaire over two or more rounds, with responses summarised and shared with participants between rounds. The Delphi method has been applied for decades across health care and other domains, particularly to develop best-practice guidelines when evidence is limited or conflicting, and to prioritise measures and generate policy recommendations [10].

### Study design and analytic methods

**Figure 1:**
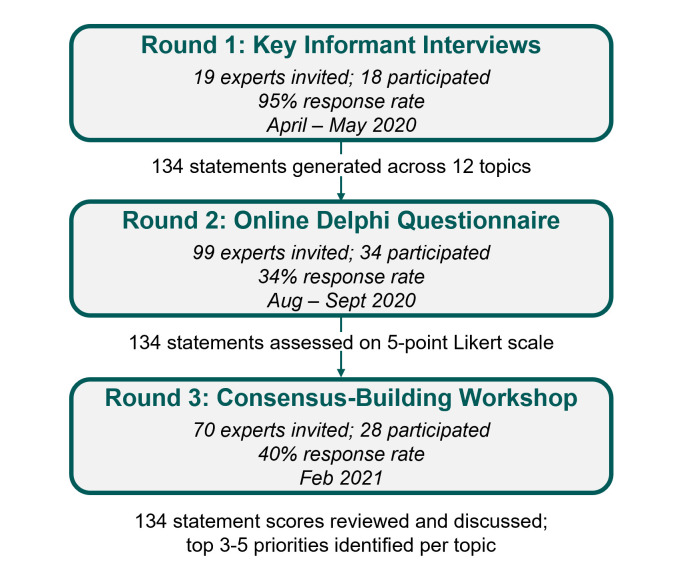
Study design and participation across Delphi rounds.

HaSET’s adapted Delphi exercise comprised three rounds (**Figure 1**). First, we conducted 18 semi-structured KIIs among MNCH experts to collect their initial opinions on challenges and needs within the MNCH research ecosystem in Ethiopia. Based on a literature review, the interview guide included questions on the strengths and challenges of existing capacity-strengthening initiatives, community engagement in research, operational infrastructure, collaborations and networks, and funding. The KIIs also covered related topics, including priority MNCH research questions, the findings of which are described elsewhere [8]. The process managers (MK, LT, BH, HT, GC) drew on their subject-matter expertise to undertake rapid thematic coding of the KII data using a framework-analytical approach [11]. The framework approach involved using pre-assigned themes to initially categorise data while also adjusting and iterating the coding scheme to accommodate emergent themes. We grouped similar responses, reframed ideas as prescriptive statements about the research environment for use in subsequent Delphi rounds and filtered ideas by feasibility and scope. This process produced 134 statements across 12 thematic topics.

In the second round, we anonymously administered an online Delphi quantitative questionnaire to a wider panel of MNCH experts, in which these 134 statements were presented for evaluation on a five-point Likert scale. Depending on the statement content, one represented ‘strongly disagree’ or ‘no importance’ and five represented ‘strongly agree’ or ‘critical/essential’. The questionnaire also allowed respondents to contribute new statements and to provide explanations of how the statements they agreed with could be accomplished.

Following this stage, we analysed questionnaire responses and calculated descriptive statistics using Microsoft Excel. For each statement, we calculated participants’ mean score ranging from one to five, reflecting the group's collective opinion. We also computed percentage agreement for each statement as the percent of responses that scored the statement as ≥4 (‘agree’ or ‘strongly agree’), and consensus was determined *a priori* to be a percentage agreement of ≥70%, a common level of consensus in other Delphi studies [12].

In the final round, we held an in-person workshop on 2nd and 3rd February 2021, in Addis Ababa, Ethiopia. On the first day, participants received printouts of all statements evaluated in the online Delphi questionnaire along with their average scores and percentage agreement. In preparation for the consensus-building discussions, participants were asked to independently rank each statement on a scale of 1–5, with one as most important, considering how these statements might translate into reality and the dependencies and challenges associated with them. The following day, participants were divided into three groups, within which they were asked to take turns sharing the statements they identified as their top priorities. A facilitator then guided the group in a consensus-building discussion using the nominal group technique to arrive at the group’s top 3–5 priorities across all statements and topics [13]. Each group was asked to reflect on key priorities within the Ethiopian MNCH context and how stakeholders could feasibly implement these priorities. Their top priorities were later presented and discussed in a plenary closing session.

### Participant selection

Participants for each round were purposively sampled based on their expertise in MNCH research and programme implementation in Ethiopia, including experts from research institutions, the MoH, regional health bureaus, non-governmental organisations (NGOs), universities, funders of MNCH programmes and research, and other organisations to advise on priority challenges and recommendations for the research environment in Ethiopia. We ensured most participants were from Ethiopia and included a few from outside of the country to provide a global perspective on Ethiopia’s MNCH research context.

### Data collection

We emailed information about the study objectives and an invitation to participate to potential participants. In April and May 2020, semi-structured KIIs were conducted by two interviewers with qualitative research expertise – one native Amharic speaker in Ethiopia who conducted phone interviews and one in-person interview, and one native English speaker in the USA (MK) who conducted interviews by phone or Zoom with USA-based participants. We drafted the interview guide in English, presuming convenience in describing technical matters, and the interviewer translated when respondents preferred to speak in Amharic. We conducted the interviews at a place and time deemed private and convenient for the participant, and interviews lasted about 30–60 minutes. We audio-recorded and transcribed all discussions in English using Microsoft Word. We also took memos during the discussions to follow emerging ideas and to refine the subsequent interviews.

For the online round, we emailed the participants invitations to complete a structured questionnaire via Qualtrics in August–September 2020. We pre-tested the questionnaire with a sample of respondents and revised to address any usability issues. We gave the participants a three-week window to complete the questionnaire, and the research team followed up by email and phone to encourage participation. Respondents took approximately 30 minutes to complete the questionnaire on average.

The in-person consensus-building workshop took place on 2–3 February 2021. We invited all Ethiopia-based experts who were invited to prior rounds, along with additional MNCH stakeholders. The first day involved prioritising research questions [8], and the research capacity-building consensus-building discussions took place on the second day.

### Ethical considerations

At all stages, we obtained informed consent at the time of data collection, and researchers described precautions to ensure confidentiality, including storing all data in a secure electronic database without personal identifiers throughout data collection, analysis, and reporting. At the end of each KII, we separately recorded participants’ background information, which was not audio-recorded.

Given the potential risk of COVID-19 transmission between researchers and participants, we developed protocols and training to minimise risk, which EPHI reviewed and approved. All data collectors were trained to follow precautionary measures, including wearing masks, maintaining physical distance, washing hands, and using hand sanitiser. Personal protective equipment was provided to all participants with instructions to always use it.

### Research team

The MoH led this research in collaboration with the HaSET Maternal and Child Research Program in Ethiopia. HaSET was founded in 2018 as an umbrella platform to build local research capacity for MNCH in Ethiopia; identify, prioritise, and conduct MNCH research; and translate evidence into policies and programmes to improve maternal and child health [7]. HaSET trains postdoctoral researchers and students and is conducting the first post-doctoral MNCH research fellowship in Ethiopia, following a learning-by-doing model where fellows are paired with colleagues at the MoH and lead research studies, conduct secondary data analysis, and translate evidence to policy briefs [14].

## RESULTS

### Participant demographics

**Figure 2:**
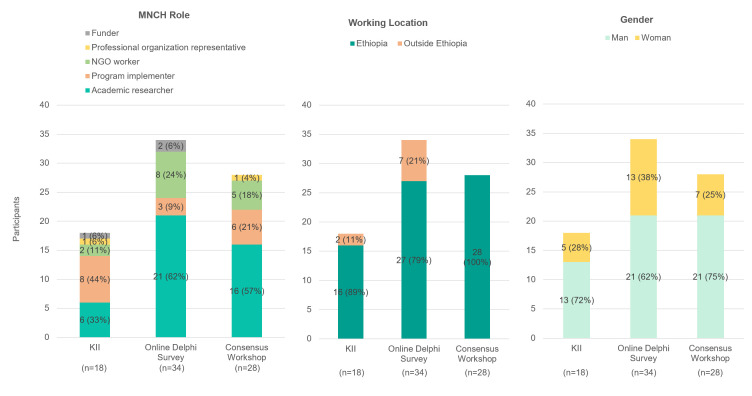
Participant demographics: MNCH role and expertise**, **working location**, **and gender.

In each round, we asked participants to self-select into the role that best describes them: academic researcher, programme implementer, NGO worker, professional organisation representative, or funder (**Figure 2**). Programme implementers, academic researchers, and NGO workers comprised most respondents across each round.

In the first round, 18 of 19 invited MNCH experts participated in KIIs – 17 by phone and one in person. Of these, 16 were based in Ethiopia and two outside of Ethiopia, while 13 identified as men and five as women (**Figure 2**). Eight were programme implementers, six were academic researchers, two were NGO workers, and one each was a funder or professional organisation representative.

For the online Delphi questionnaire, 34 of 99 invited experts participated, yielding a response rate of 34%. Most respondents came from academia (62%), 79% were based in Ethiopia, and 62% identified as men. Of all respondents, 21 were academic researchers, and no professional organisation representatives responded to the questionnaire.

At the in-person workshop, 28 of 70 invited experts participated in the consensus-building discussions. All participants worked in Ethiopia, and 75% identified as men. The majority (57%) were academic researchers, with 21% programme implementers and 18% NGO workers; no funders participated in the workshop.

### Key themes from the KIIs

**Figure 3:**
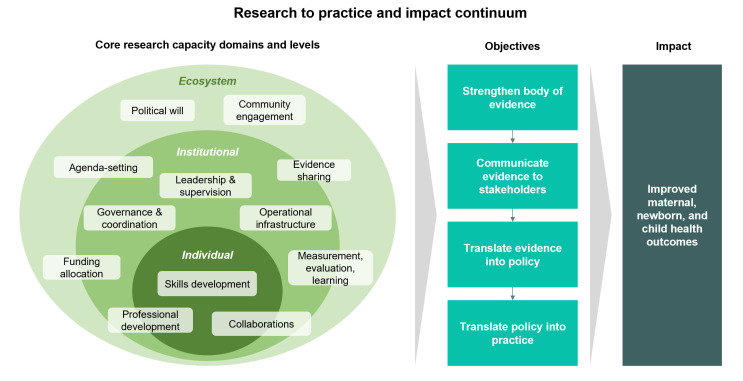
Research to practice and impact continuum.

Participants identified a variety of gaps in the research-to-policy lifecycle, including in educational and training content and structure, availability of local experts and mentors, community engagement in research, and the relationship between research and policy priorities and needs. We synthesised the themes surfaced from KIIs into a conceptual model (**Figure 3**) of the research-to-practice and impact continuum, highlighting key capacity components and domains where gaps were identified and future interventions may be targeted. Namely, research capacities at the interrelated levels of the individual, institution, and ecosystem contribute to the objectives of strengthening the body of MNCH evidence, communicating it to various stakeholders, and translating it into policy and practice – with the ultimate goal of impacting MNCH outcomes.

In total, 134 statements were identified from a thematic analysis of the KIIs and categorised into 12 topics spanning this continuum (**Table 1**). Below are key themes from the KIIs organised by each of these 12 topics, and we provide a selection of illustrative quotes for each theme (Appendix S1 in the **Online Supplementary Document**).

**Table 1:** MNCH research environment topics evaluated

Research environment topic	Likert scale used	Total statements, n (%)
Improving the institutional MNCH research environment	Strongly disagree – Strongly agree	7 (5)
Improving MNCH research agenda-setting	Strongly disagree – Strongly agree	9 (7)
Research training priorities	No importance – Critical/essential	15 (11)
Improving academic research capacity and training	Strongly disagree – Strongly agree	11 (8)
MNCH evidence sharing	Strongly disagree – Strongly agree	4 (3)
Improving research collaborations for MNCH	Strongly disagree – Strongly agree	5 (4)
Metrics of success for an MNCH research network	No importance – Critical/essential	5 (4)
Enablers of the translation of MNCH research to programme and policy action	No importance – Critical/essential	14 (10)
Importance of engaging community actors	No importance – Critical/essential	11 (8)
Enablers of community-based research	Strongly disagree – Strongly agree	24 (18)
Operational infrastructure improvements for MNCH research	No importance – Critical/essential	17 (13)
Priorities for MNCH research funding	Strongly disagree – Strongly agree	12 (9)

MNCH – maternal, newborn, and child health

### Improving the institutional MNCH research environment in Ethiopia

Improving the institutional MNCH research environment in Ethiopia is crucial to developing the country's research capacity. Overwhelmingly, respondents described poor coordination between the various entities and levels of governance contributing to research – from the MoH, which oversees regional health bureaus and facilities, and the Ministry of Education, which oversees approximately 50 universities, to other government agencies, programme implementers, academics, and policymakers. There is no clear pathway for setting research priorities, synthesising research findings, or translating findings into policy, and transparency is lacking about which actor is undertaking which research. While the MoH develops research plans and guidelines, their socialisation among relevant stakeholders is sometimes lacking, partly due to highly bureaucratic processes and high leadership turnover. One respondent described, ‘because of the bureaucracy, many partners could not get what they want or finish what they want to on time, or they may return or discontinue because of the leadership problem’. Respondents expressed that many intelligent and capable Ethiopians are held back by weak institutions, poached by international organisations and the private sector, or lost to ‘brain drain’.

A need to strengthen institutions and improve communication throughout the health research ecosystem, including with lower-level facilities and regional universities, is evident. Strengthening regional universities' capacities is seen as an important step in improving the institutional MNCH research environment. One respondent described national universities as better resourced and having more contact with international researchers and the global literature, while regional universities that were established to serve local needs ‘are totally cut off from what is happening in the rest of the world: they don’t know, and nobody tells them, and the faculty teach courses from outdated textbooks’. Appetite is strong, however, for better coordinating RMNCH research, including through the design of national and regional research hubs and modelling RMNCH networks after other successful disease-specific research networks.

### Improving MNCH research agenda-setting

The prioritisation of critical MNCH research questions does not take place regularly or transparently. While the MoH Maternal and Child Health Directorate has a Research Advisory Council (RAC), the voluntary nature of the RAC was described as a challenge to its effectiveness, and its priorities are not widely adopted.

Multiple respondents also described politics as a challenge in setting research priorities. Ethiopia is a diverse country with a history of ethnic tensions manifesting in political divisions, and the needs of groups that are not in power do not receive as much health system investment or research priority. A respondent also described difficulty in obtaining permission to objectively evaluate the government’s flagship health programmes, such as the health extension worker and women’s development army programmes. Moreover, multiple respondents described how reliance on donor funding results in research prioritising donor-driven agendas over local health needs.

Respondents characterised a strong research agenda-setting process as one that addresses priority health needs for the country, following a systematic, inclusive approach to guide research investments while also allowing for flexibility to accommodate emerging topics and priorities. Historically, drivers of research agendas have included global priorities as well as academic and professional gain, but ‘the objective of the research (should be) problem identification and analysis for the improvement of community health problems, not only creating new things and knowledge’, as one respondent described. A systematic approach should prioritise input from the lower levels of the health system and from communities themselves.

### Research training priorities

Technical capacity gaps were identified throughout the research process, from the formulation of the research question and study design to translation. Specific areas for improvement highlighted include methodological rigour, understanding and overcoming data quality limitations, using qualitative methods, writing proposals and manuscripts, and complex modelling and statistical analysis.

Beyond training researchers, respondents highlighted the need for educational institutions and programmes to better train policymakers to use evidence in decision-making, and for health care professionals to understand the importance of research in improving practice.

### Improving academic research capacity and training in Ethiopia

Many students are interested in research but are unable to develop to their full potential within the current academic environment. One respondent described, ‘The quality of research currently conducted by the academic institutions is lower in quality than the programme research. This is due to low-quality education’. Specific challenges in the academic environment include the training gaps detailed above, the privileging of external evidence and experience over local knowledge, a lack of both formal and informal mentorship, and the dearth of practical, experiential learning opportunities. For example, apprenticeship programmes that place students within different parts of the health system, NGOs, and other institutions to identify potential research opportunities would be valuable in both driving needs-based research and helping students gain critical hands-on experience.

Multiple respondents described an overarching need for academic institutions to foster a stronger culture of critical thinking and problem-solving: ‘Our universities are just producing people. We teach by copying something written by somebody... Ideally universities are intellectual centres, developing ideas, critiques and arguments, being sceptics, but now it is not so’. Within this environment, research undertaken to meet academic requirements does not always address priority health needs.

As a result of these constraints, among other sociopolitical considerations, ‘brain drain’ was described as common, highlighting the need for Ethiopia to better retain its research talent domestically. One respondent even recalled an instance when external actors provided capacity-building support to universities but ended up poaching local researchers. As local researchers with skills in high-level modelling and complex statistics are in short supply, they are often in high demand and overstretched.

### MNCH evidence sharing in Ethiopia

Data and evidence sharing was characterised as very poor, relating to both structural and cultural challenges. Structurally, mechanisms for sharing data and knowledge are lacking, including between researchers themselves, between researchers and health care providers, and with broader audiences. For example, universities may host research symposia, but these are full of scientific presentations and not communicated to the level of other audiences, as one respondent described. Sharing is largely the result of individuals’ own initiative rather than facilitated by structures or guidelines: ‘Only if you personally know a research professional are you able to consult and get information... Most of the time, we perceive that information is power, and we never need to share. It is an individualistic perception’. Another respondent described clinicians' reluctance to share data with public health researchers. Sharing may also be constrained by political pressure, with one respondent describing, ‘We are experienced in hiding the research findings that are not suited to the political system. This causes delay in the provision of solution to the problems’.

### Improving research collaborations for MNCH in Ethiopia

Generally, respondents described a weak culture of collaboration within the MNCH research environment, which, given limited research resources, is seen as a misuse of those resources. Resource scarcity can also foster a sense of territorialism among organisations, leading them to be reluctant to share funding with others. While collaboration between academic and non-academic institutions is increasingly encouraged, one respondent expressed concern about institutions' and bureaucratic systems' ability to effectively manage large collaborations.

Within existing collaborations, branding and ownership can pose challenges; roles, responsibilities, and benefits to each party should be equitably distributed and clearly delineated upfront. A primary challenge described was around data hoarding and the need for open sharing agreements among partners: ‘In collaboration, there should be transparency. The data are a country’s resource. Sometimes funders give money and do not allow the data to be used by local researchers’.

While involving multiple stakeholders (academia, implementers, policymakers) in research is viewed favourably, each stakeholder’s objectives for the research and its use are often unique, which can lead to disagreements on workflows and deliverables. Understanding each partner’s objectives and aligning at the start on a common shared goal can help prevent this friction. Additionally, respondents described the need to involve a greater diversity of stakeholders in research collaborations, including valuing regional universities on par with national universities. There is also a desire for more South-South partnerships between Ethiopia and other countries, which may help with competitiveness for grants while allowing local researchers to learn from and exchange with others.

Challenges, successes, and opportunities were also described for international collaborations with high-income country partners, in particular. When external partners have done the work to understand the local context before arriving, are respectful of local research stakeholders, are responsive to challenges that arise during implementation, and include long-term, progressive capacity strengthening opportunities, such collaborations are viewed favourably. On the other hand, when local researchers are relegated to data collectors rather than true partners, such collaborations are extractive.

Many respondents described how capacity strengthening within international collaborations should be conducted. Collaborations should be long-term, enabling external researchers to better understand and address local needs, while affording local researchers greater opportunities for mentorship, given the dearth of local mentors, as described above. One high-income country respondent described, ‘If you stick it out over the long run, you’ll have a much better chance to improve capacity. For the American, Canadian, or European researchers, over time you’ll learn the way you wanted to do things made no sense, and you’ll become humble and respond better to local needs... Short-term training, even if cutting-edge in some respect like teach some new methods, won't do as much as if it's long-term’. Accordingly, many respondents would like to see cumulative capacity-strengthening training and activities, with cohorts of researchers supported to progress through the full research lifecycle, rather than receiving isolated training. Training should ideally be experiential, with the ability to put new learnings into practice immediately, and tailored to identified gaps in research capacity.

### Metrics of success for an MNCH research network

For MNCH research and networks, respondents overwhelmingly characterised success as contribution to the improvement of Ethiopia’s priority health concerns, or as one respondent put it, ‘collecting data that matters’. Metrics to assess the contribution of research to health improvement should consider the full spectrum of problem identification, from generating, testing, and scaling solutions to the range of research capabilities required at each step. Specific measures described include the quality of research and the availability of research findings and data to different audiences, informing not only the academic evidence base but also day-to-day decision making in communities; diverse representation in MNCH research networks, including regional stakeholders, all related health care disciplines (obstetrics, paediatrics, and more), policymakers, and communities; and networks’ sustainability and ability to incubate new research leaders. ‘In the long term, a successful research programme would have a plan for succession and mentorship of bringing junior investigators up and gradually taking over some leadership roles’, one respondent described. Ideally, successful networks will also generate MNCH knowledge to inform not only local but global practice. Critically, these success measures target the quality and impact of the research, whereas respondents noted that the current system often prioritises the quantity of research produced, particularly for academic and professional gain.

### Enablers of the translation of MNCH research to programme and policy action

Overall, the ineffective translation of research into policy in Ethiopia was described as a major gap inhibiting MNCH progress, largely due to weak connections among academics, clinicians, policymakers, and implementers. A divide was described between clinicians and public health researchers, in which clinicians may lack the time and methodological expertise to conduct research, while researchers may lack the subject matter knowledge to conduct appropriate studies, and little structure or incentive exists for these stakeholder groups to collaborate.

On the policy front, many respondents described insufficient capacities on the part of policymakers to effectively interpret and synthesise research to inform policy, including assessing the strength of the body of evidence and identifying the contribution of specific interventions. As one respondent described, ‘Sometimes Ministry people don’t have a good understanding of research – what it means and what it requires to do high quality research – so that can make it also very difficult to involve them, but also to help them understand the findings’. Others described policymakers relying more heavily on external evidence than on locally generated research to develop guidelines and policies. Research is not always communicated effectively to policymakers or across levels of government, policymakers are constantly balancing multiple priorities, and institutional memory is lost with high turnover among policymakers – all of which can inhibit research translation efforts.

Finally, resource constraints can hinder translation, particularly when research infrastructure and support are made available for a research project but are not readily available in health facilities where the studied intervention would later be implemented.

However, momentum toward greater research coordination, uptake, and utilisation has been increasing in recent years. The RAC was created as a platform to inform policy with research, and while its volunteer composition was described as a challenge in its effectiveness, respondents describe it as a strong step in the right direction. Research translation was also described as effective in specific areas, such as the community health workforce and newborn care.

To improve research translation, respondents described opportunities, including having a dedicated unit to synthesise research and liaise between the MoH and research institutions. Respondents described a need to strengthen skill sets to effectively package and communicate research findings for different audiences, including policymakers, programme implementers, and communities – as EPHI has been doing with the creation of policy briefs – to ensure that research is not ‘left on the shelf.’ Bringing MoH and programme implementers into research as partners from the design stage was also described as critical to ensuring buy-in and translation.

### Importance of engaging community actors

The importance of engaging communities in research was widely recognised among respondents, who believe that communities’ lived experience affords them greater familiarity with the research context, ability to characterise their health challenges and potential solutions, and ability to interpret research findings within the local context. While this importance is recognised and codified in some respects, meaningful community engagement is variable in practice. Plans to disseminate research findings to the community are often included in proposals to obtain ethical clearance, but not actually realised. Cost was cited as a primary factor limiting community engagement, as returning to communities after research completion is resource-intensive, and this additional expenditure can be difficult to justify in an already resource-constrained environment, especially when the challenges being researched are related to poverty. Moreover, some communities do not trust or engage with the health care system, complicating researchers’ ability to reach these groups. When community engagement does occur, respondents have found it highly beneficial to the overall research process and to communities’ sense of trust and ownership, and there is a strong desire to further institutionalise community engagement.

### Enablers of community-based research

Respondents emphasised the need to engage communities early – from design through dissemination – to increase their sense of ownership and the likelihood of translation of the research into action. Inclusive, co-creation approaches involve engaging communities in identifying and prioritising research questions, shaping the research plan, and identifying barriers to reaching the research goals. Establishing formal mechanisms, such as community advisory boards, can facilitate this, particularly for longitudinal studies. Guidelines and trainings can also bolster researchers’ skillsets in appropriately engaging communities – for example, in ensuring materials and communications are translated to local languages and identifying the appropriate mix of community voices to engage, including women who do and do not seek care through the formal health system, religious and clan leaders, youth, and men and other family members. While it is more resource-intensive, respondents emphasised the need to prioritise minority-language populations and those in hard-to-reach geographies to ensure their representation in MNCH research and, ultimately, in the policies and practices that should benefit them. Acknowledging communities’ contributions to research should be prioritised as well, including through the provision of learning and training opportunities. More than acknowledgement and dissemination of findings, though, communities want to see and experience changes resulting from the research and not just provide data. While the ability of a study to contribute to tangible change is not fully within the research team’s control, demonstration of a long-term commitment to the community is critical, and respondents described several ways to strengthen the research-to-translation pathway (see above).

### Operational infrastructure improvements needed for MNCH research

Respondents described a range of deficiencies in operational infrastructure, from physical needs, such as office space and laboratory capacity, to software and institutional constraints that limit the types and quality of research they can conduct. Internet connectivity can vary, and many institutions lack access to paid journals and databases, leaving researchers to request articles from their contacts abroad. Data quality, standards, and management were described as significant obstacles, with the absence of standards for unique identifiers, absence of clinical and patient databases for research use, and poor quality of data from clinical and medical records: ‘Ethiopia is one of the most constrained in terms of resources and health facilities; it’s hard to have high quality research next to poor quality (records and) clinical care’. Respondents described information technology capacity as very limited, with data collection software and devices available on an ad hoc basis if supported by project funds, and institutional review board processes were described as cumbersome. Overall, research institutions need stronger financial and grant management systems to handle projects more efficiently and on a larger scale.

Together, these obstacles create an environment that cannot readily support more complex research undertakings: ‘When we compare ourselves with other West and East African countries, we lag in clinical and vaccine trials. The legal framework in our country does not open for this. We are conducting clinical trials through other countries’ laboratories like the USA, Europe, or South Africa, where samples and data are shared to others’. These constraints also limit the experiential learning opportunities that researchers are afforded: without access to cutting-edge journals, for example, students have less opportunity to conduct literature and systematic reviews.

### Funding priorities for MNCH research

The challenges described above depict a resource-scarce environment for MNCH research in Ethiopia, with a heavy reliance on donor funding that does not always align with local research and RSC priorities. Moving forward, respondents emphasised that a commitment from donors to funding longer-term partnerships will be critical to begin addressing many of these challenges, including fostering collaborations of greater trust, deeper community engagement, stronger local leadership structures, and progressive capacity-strengthening opportunities. This includes funding overhead to strengthen research institutions’ infrastructure to promote a stronger enabling environment for research and RCS. It also includes developing long-term platforms to support researchers’ growth, from academic and technical training to experiential learning in running high-quality, complex research studies. Other ideas suggested include diversifying funding among local researchers, as funding may repeatedly go to the same individuals who are considered the ‘go-to’ for certain topics, making it difficult for other researchers to access external funding opportunities.

### Online questionnaire results

Of the 134 statements, the average percentage agreement was 87% (range = 37–100). Of all, 122 statements reached consensus with ≥70% agreement (Appendix S2 in the **Online Supplementary Document**) and 14 statements received full (100%) agreement among participants (**Table 2**).

**Table 2:** Statements with full agreement (100% of respondents agree or strongly agree), by topic

Topic	Statement	Agreement, %
Improving MNCH research agenda-setting	Agenda setting should always be through an iterative and inclusive process.	100
Improving MNCH research agenda-setting	Research approval process should prioritise the relevance of a research project in addressing priority MNCH problems over personal or external motivations.	100
Research training priorities	Data analysis and interpretation.	100
Improving academic research capacity and training	Teaching should prioritise local research experiences, not only examples from European and other countries.	100
Improving academic research capacity and training	Medical and nursing schools should educate students on the importance of research and on research methodologies.	100
MNCH evidence sharing	Universities should facilitate a forum for local evidence-sharing.	100
Improving research collaborations for MNCH	Collaborations should have a substantive capacity-building component for junior researchers and universities.	100
Improving research collaborations for MNCH	Collaborations should be long-term partnerships involving long-term mentorship of junior researchers over their career.	100
Improving research collaborations for MNCH	Collaborations should be designed to bring public health experts and clinicians together in multidisciplinary teams.	100
Importance of engaging community actors	Women (community actors important to engage).	100
Importance of engaging community actors	HEWs (community actors important to engage).	100
Enablers of community-based research	Researchers should share back research findings with communities during or after the research has completed, such as through a day discussing and validating the findings and addressing any community concerns.	100
Priorities for MNCH research funding	MNCH research funding should prioritise knowledge translation and management.	100
Priorities for MNCH research funding	International MNCH research funding should prioritise longer-term visions and investments.	100

MNCH – maternal, newborn, and child health, HEW – health extension worker

Among statements with the strongest agreement were designing research collaborations to bring public health experts and clinicians together in multidisciplinary teams and to provide substantive, long-term capacity-building opportunities for junior researchers and universities; setting research agendas through an iterative and inclusive process and by prioritising the relevance of the research to key MNCH needs; and funders prioritising knowledge management and translation as well as longer-term investments.

### Expert group consensus-building workshop results

Each of the three discussion groups identified different statements as their top five priorities, reflecting the themes and compositions of their groups (**Table 3**). Of the 15 priorities identified across groups, all had scored an average of ≥4.1 of 5 and reached consensus (≥70% agreement) in the online Delphi round, and they included statements across all 12 topics.

**Table 3:** Top five priorities for each discussion group

Topic	Statement	Discussion group	Agreement, %	Average score
Improving MNCH research agenda-setting	Agenda setting should always be through an iterative and inclusive process.	Group 1 - maternal health	100	4.7
Improving the institutional MNCH research environment in Ethiopia	The relationship between academic universities and regional health bureaus should be strengthened to better prioritise research questions and translation of evidence to policy.	Group 1 - maternal health	97	4.8
Research training priorities	Translation of research to policy.	Group 1 - maternal health	97	4.7
Research training priorities	Research methodologies and study design.	Group 1 - maternal health	94	4.5
Improving academic research capacity and training in Ethiopia	More local PhD and MSc programmes are needed.	Group 1 - maternal health	70	4.1
Improving research collaborations for MNCH in Ethiopia	Collaborations should be designed to bring public health experts and clinicians together in multidisciplinary teams.	Group 2 - newborn health	100	4.9
MNCH evidence sharing in Ethiopia	MoH should facilitate a forum for local evidence-sharing (*e.g.* at the regional, district, and zonal levels).	Group 2 - newborn health	97	4.5
Metrics of success for an MNCH research network	Number of research studies that are incorporated into policy briefs and implemented.	Group 2 - newborn health	94	4.6
Enablers of the translation of MNCH research to programme and policy action	Research being motivated by improving specific programmes.	Group 2 - newborn health	93	4.2
Importance of engaging community actors	Community/kebele leaders.	Group 2 - newborn health	93	4.3
Priorities for MNCH research funding	MNCH research funding should prioritise knowledge translation and management.	Group 3 - health systems	100	4.7
Priorities for MNCH research funding	MNCH research funding should prioritise evaluating the implementation of evidence-based interventions.	Group 3 - health systems	96	4.5
Enablers of community-based research	Investments in community-based research should be linked to programming for those communities involved in the research.	Group 3 - health systems	93	4.5
Enablers of community-based research	There should be protocols and guidelines for researchers to appropriately engage communities.	Group 3 - health systems	93	4.4
Operational infrastructure improvements for MNCH research	Improving access to and quality of IT centres.	Group 3 - health systems	87	4.3

MNCH – maternal, newborn, and child health, MoH – Ministry of Health, IT – information technology

## DISCUSSION

RCS efforts in the health system are important to build national capacity to generate robust, innovative, and contextually relevant research that can inform policy and practice to improve population health. The challenges characterising the MNCH research environment in Ethiopia are clear, with the Delphi statements strongly echoing themes and recommendations across the RCS literature on Ethiopia and low- and middle-income countries.

All seven statements on ‘Improving the institutional MNCH research environment’ reached consensus in the online questionnaire, underscoring the need for stronger governance and clearer delineation of roles and responsibilities across research institutions, including the MoH, RAC, EPHI, and the Armauer Hansen Research Institute. Strong research leadership and coordination are critical enablers of a healthy MNCH research ecosystem. These can be promoted through embedding research leadership capacity development into institutional strategic plans, cultivating political will and recognition of the value of local research, and developing leadership in both research and research management [15].

The hierarchy of evidence suggests that decision-making should prioritise methods with the least bias, such as systematic reviews, meta-analyses, and randomised controlled trials, rather than relying solely on expert opinion or anecdotal evidence. Health system policymaking must also weigh factors such as feasibility, political context, and cultural considerations when translating evidence into policy and practice. For agenda-setting, the consensus emphasised the need for a clear mechanism to guide how evidence is solicited and used in problem identification and priority-setting so that existing research can be incorporated systematically into the vetting process.

Increased investment in health research from African governments would allow them to assume ownership of research agendas and sustain existing capacity-building efforts. Funding mechanisms should be shaped with input from diverse stakeholders – including investigators, policymakers, and community members – to ensure alignment with local needs. The 14 statements that achieved full agreement reflected respondents’ strong preference for an inclusive, iterative agenda-setting process that prioritises key MNCH issues. Similarly, the large number of statements on community-based research highlights participants’ recognition of the importance of engaging communities in shaping, implementing, and benefiting from research.

For any research capacity-strengthening to have a long-term impact, long-term funding of programmes is essential. The themes prioritised in this study mirror those emphasised by Scott et al [16], who stressed the importance of institutional roles in training, funding, and fostering connections between researchers and policymakers. Many of the highly endorsed statements also align with Brodén's [17] recommendations on the quality of higher education, agenda-setting, end-user engagement, research translation, and systematic approaches to research development.

During the consensus-building workshop, expert groups prioritised statements on strengthening multidisciplinary collaboration between clinicians and public health experts, reinforcing partnerships between academic institutions and regional health bureaus, enhancing translation of evidence into policy and practice, prioritising knowledge translation and management in research funding, facilitation of a forum for local evidence-sharing (*e.g.* at the regional, district, and zonal levels) by the MoH, prioritising evaluating the implementation of evidence-based interventions, and linking investment in community-based research to programming for those communities involved in the study. These priorities echo Gaym’s recommendations to strengthen Ethiopia's national health research agenda [18].

While all statements are important to building a successful research environment, the 15 priorities identified through group consensus (**Table 3**) provide a clear pathway from capacity building to evidence generation, policy uptake, and evaluation. Bowen and Zwi similarly emphasised the interconnectedness between sourcing evidence, using evidence in policymaking, and building implementation capacity [19].

Incentive mechanisms to support community members’ participation in research, together with national guidelines on community engagement, were also highlighted. These recommendations align with Vanderslott et al, who described three pillars of community engagement in health research: communication to build relationships, generating contextual knowledge, and sustained learning over time [20].

The discourse on ethical and equitable research partnerships is rapidly growing, and while evidence on effectively implementing RCS programming remains limited, frameworks and indicators for assessing RCS effectiveness are emerging and should be tested across implementation settings. For example, the Centre for Capacity Research and the African Population and Health Research Centre developed practical guidance and indicators for RCS evaluation [21], and the insights surfaced and prioritised in this study align strongly with their recommendations. Global efforts to standardise measurement of research capacity, including the World Health Organization Global Observatory on Health R&D’s efforts to establish a global set of harmonised and agreed-upon indicators to assess national health research systems [22], will further contribute to an understanding of RCS over time that should be contextualised and supplemented by local knowledge.

### Limitations

Several limitations need to be addressed. The gender balance was skewed across rounds, with most respondents being men (**Figure 2**). The sample for the in-person workshop was smaller than that of the online questionnaire and more restricted, due to COVID-19 travel restrictions, potentially omitting key voices from those discussions. Finally, representation of perspectives from different organisational types was limited and variable across the rounds of data collection: no professional organisation members participated in the online questionnaire, and no funders attended the in-person workshop.

A limitation of the Delphi method is the lack of established standards for reporting Delphi studies or validated parameters to evaluate their quality, though efforts are under way. The guidance on Conducting and REporting DElphi Studies is a useful tool, initially developed for the context of palliative care [23], and in 2023, RAND published the Delphi Critical Appraisal Tool checklist to guide both Delphi design and reporting [10], though it was published after we collected the data.

### Strengths

We provide an in-depth characterisation of the MNCH health research environment and opportunities to strengthen health policymaking in Ethiopia. Through the participation of experts with different roles across research and the use of robust iterative methodologies, we identify and characterise the research environment across critical dimensions, spanning community engagement, research institution activities, and research infrastructure. We also provide specific opportunities for involved stakeholders to strengthen the identified areas of need to produce high-quality research and translate evidence into policy.

Given the lack of robust evidence on effective research capacity-strengthening efforts, which are often complex and heterogeneous [24], expert consensus methods provide a valuable opportunity to understand research environment needs and priorities. To do so, this study invited participants with deep experience across the MNCH research landscape in Ethiopia to contribute both their expertise and their lived experience to developing priorities for future initiatives.

The Delphi method is flexible and can be tailored to specific research objectives; its strengths include anonymity, iteration, controlled feedback, and consensus [10]. Anonymity removes typical biases of in-person interaction, such as conformity to dominant views and groupthink. It may also encourage participants to be more honest in their assessments, particularly when discussing sensitive issues with other participants from different backgrounds. The controlled feedback, with summary data analysed and presented to participants between rounds, allows individual members to observe trends and amend their response, and this pressure for convergence can be seen as both a weakness and a strength.

### Application of results

Understanding challenges in the existing research environment will enable more informed activities and stronger research networks that address local priorities. The opportunities highlighted through this study will be relevant for national and international advocacy, curricula development, and the implementation of principles to guide partnerships and agenda-setting for a variety of actors. HaSET, specifically, is embedding these insights into its ongoing RCS programming and developing evaluation strategies to inform future learning [14]. As a result of this work, the MoH is committed to fostering multidisciplinary collaboration, bringing together experts in public health, clinical health, and policy-making to strengthen MNCH research and ensure policy integration. Recommendations for achieving high-quality, impactful research needed to inform key polices include creating an environment that fosters intellectual curiosity, linking researchers and policymakers throughout the policy life cycle, and identifying and prioritising policy-relevant research questions together. Actionable suggestions for further work include investing in a culture of evidence and rigour, and securing policymakers' commitment to implement and scale evidence-based findings into policy and practice. Leveraging HaSET’s existing and expanding partnerships in Ethiopia, we anticipate that the results of this modified Delphi exercise will influence not only MNCH research over the coming years, but also policy and programme decisions with the potential to reduce the MNCH-related disease burden over time.

## Additional material


Online Supplementary Document
