# Supplementary material for: Prioritising opportunities to strengthen the maternal, newborn, and child health research ecosystem in Ethiopia: a Delphi exercise
Source: J Glob Health. 2026 Jan 23;16:04001. doi: 10.7189/jogh.16.04001 (PMC12828438; doi:10.7189/jogh.16.04001)
Supplement: Online Supplementary Document [file jogh-16-04001-s001.pdf]

Supplement to: Taddesse L, Korte M, Hunegnaw B, Teklie H, Bekele D, Tolera G, Zelalem M, Chan G. Prioritising opportunities to strengthen the maternal, newborn, and child health research ecosystem in Ethiopia: a Delphi exercise. J Glob Health. 2026;16:04001.

Appendix S1. Illustrative quotes from KIIs

Topic	Illustrative quotes
Improving the institutional MNCH research environment	"There are structural challenges – poor coordination between the Ministry of Education and FMoH. Under the Ministry of Education there are about 50 universities and under MoH there are health bureaus and facilities. Between this is no intentionally established structure for how to collect and use evidences generated by research institutions."
	"There is good commitment to collaborate and to work transparently at FMoH level. At FMoH level, they know what type of evidence they need for specific programing. At the regional level, it's somehow good. But at lower level there is weak leadership commitment, capacity, and collaboration."
	"The main gaps observed between research agencies in FMoH and RAC is lack of coordination even with the directors found within the Ministries. RAC evolved for RMNCH research, but EPHI should support the RAC process for all programs. EPHI and FMoH should work jointly on the research, and full assignment of research leadership should be provided to EPHI."
	"Because of the bureaucracy, many partners could not get what they want or finish what they want to on time, or they may return back or discontinue because of the leadership problem. There are disappointments because of the bureaucracy… Since we are poor, there is high corruption, and the bureaucracy might be to cut the corruption. To counteract the corruption, the system taken in place is so stringent. It is not only in research; it is a nationwide problem."
	"Another thing that happens is in the public sector with a really good administrator, that person will be poached by a private sector company, because the private sector company gives better benefits and better salary, so why not? There's lots of intelligent and creative Ethiopians, but somehow they don't stick around in the Ethiopian institutions that might really be able to change things."

	"The national universities have much more contact with international researchers and the global literature, but those local universities are totally cut off from what is happening in the rest of the world. They don't know, and nobody tells them, and the faculty teach courses from outdated textbooks. You might not have many faculty members with even a doctorate in some departments."
Improving MNCH research agenda-setting	"Research is done for sake of research: a student to graduate and the instructor to [obtain promotion], not for solving problem."
	"Objective of the research [should be] problem identification and analysis for improvement of community health problem, not only creating new things and knowledge."
	"If we are thinking as a system, we need to establish independent system which goes to the lower level, for instance as that of the NIH of America. It develops research agendas, control research outcomes. It may take time and needs resources but the most effective ways. Until then, we need to strengthen research capacity, integration of junior with senior and those with less experience with ones who have better skill, and also improving communication to know who is doing what. Sharing experience, involving students on mega projects of the universities."
	"As a system, there is no structure that sets research agendas, priorities, and also synthesizes the available research…. No one knows who is doing what."
	"The biggest challenge is politics. One problem is the ethnic tensions in the country. The needs of the groups who are not in power don't get as much attention. In rural parts, you'll find in one area the government will invest lots of money into improving infrastructure and building new health facilities and will bring foreign guests out to see how good things are…. It's hard for us to appreciate the overall situation in rural Ethiopia, because some areas we're allowed in and we get to see places where things are going well, but there are other areas with repression, and with repression you have worse maternal and child health outcomes, and we don't get to see that. Certain ethnic groups are subject to a lot of repression, and we don't learn how they're doing."
	"There isn't really space to say that what the government is doing isn't working…. If you critically analyze and say it doesn't look like [government initiatives are] working well, that

	doesn't go over very well, and you might not get permission to do that research. It's a point of pride for the government, so you don't have full ability to critically analyze them."
Research training priorities	"Most of Master's researches on MCH is not problem solving, really, I don't think so. It is a mass production."
	"[For clinicians] there might be a problem in understand [research's] importance. Therefore, it is crucial in providing awareness on the benefit of their engagement in the research. Institutions like medical, nursing school are responsible to inform and teach about it."
Improving academic research capacity and training	"Institutional capacity building – there should be PhD and MSc programs in home country and local institutions [rather] than taking a person for five years to Canada or somewhere else."
	"Brain drainage is the main challenge – external supports as capacity building come to our universities but finally the took our researchers. It was intended to building the capacity but ends in capacity weakening."
	"The issue that comes up over and over again is mentoring. Especially doctoral students but also masters students, for the faculty to give them feedback on proposals, on manuscripts they're working on, help them find their way in a complex environment; that seems to be lacking. Some are exceptions who do miraculous things and go out of their way to help students, but you have many more faculty who don't make it their priority, or they themselves never got mentored so don't understand why this new generation of students may be so bold as to demand mentoring."
	"There are clear technical gaps, but the capacity building should not be given in a blanket, it should fit for purpose. If there is a grant writing call people need to take grant writing training and similarly for data analysis. Capacity building need to be given for the right people with right intended purpose."
	"There are no shortage of really good Ethiopians, but a lot of them are outside Ethiopia. In New York and Geneva and Washington, there are tons who left Ethiopia at this point, likely because of politics. Does Ethiopia need more external support and mentoring from people like me, or does Ethiopia just need some way to retain the qualified Ethiopians in Ethiopia? I think

	ideally we do a lot more if we retain the qualified Ethiopians in Ethiopia. In that regard I see Uganda and Kenya as being more successful."
MNCH evidence sharing	"There is high staff turnover and this resulted loss in institutional memory. The research coordination is low in terms of data sharing and over all due to limited knowledge and capacity."
	"Previously, public health professionals do more research than clinicians do. Young public health professionals are also promoted to associate professor level and in the near future many young professionals become full professors. But clinicians who served for longer years did not promote to these levels because they did not do research. Now they considered as they are over taken by public health, thus they do not allow others to collect data."
Improving research collaborations for MNCH	"There are so many different partners local, international, professional association, implementing partners, universities, all partners have the intention to do all activities by themselves…. The resource available for research, intervention and service delivery is very limited. So, if people have the intention to collect all resources and to do all the activities by themselves it creates misuse of resources."
	"From the beginning to the end, the research should be conducted collaboratively by sharing. We have a collaborative research with universities, however the interest of us and universities is completely different. Universities needs for publication; we need evidence for program environment and improvement… This causes disagreement as a result of the deliverables in the project. The academic institutions and programmers should work in equal understanding of the context ahead of receiving research grant. The researcher viewed research from research perspective only. We implement the program; it is so complex. Understand each other is very important."
	"The culture of partnership is weak in our country."
	"Here, we are so behind to have these types of collaboration [as seen with international actors]. The whole bureaucracy might not allow; they provide small grant. Even if they collaborate to do large scale study with another institution, I do not know how the system provide grant for such type of research because there is no structure that support this types of collaborative research."

	"There should be a discussion between junior and senior researchers. In addition, the room should be created juniors for challenging senior researchers."
	"In partnership projects, the role of local universities should not be data collectors; there should be a capacity building component including providing opportunists for Master's and PhD programs."
	"From the design stage there should be agreed data sharing mechanism. For example, a means sharing data to the public. Most of the time there is a data sharing agreement among the partners. We do not have a practice of this."
	"By providing two days and two-weeks workshop, you cannot make a person researchers, I do not believe on that. It is a skill that you learn by doing, doing and doing."
	"The role of local universities should not be data collectors; there should be a capacity-building component including providing opportunities for masters and PhD programs."
Metrics of success for an MNCH research network	"A work become complete when it passes through different stages, problem identification, finding the solution, applying the solution, scaling up the better solution and… improvement of health status. Indicators should consider all these steps."
	"For me, success is all about contribution…. For the academics by contribution they made for science, scientific implication. We ask our PhD students three questions: reliable, originality, contribution; contributions for science and community. Public health or clinical contribution, the contribution for the improvement of health service, and community health."
Enablers of the translation of MNCH research to program and policy action	"There is no bridge which brings the health system and academics together… The contribution of academic for policy, program and strategy has to be examined."
	"Poor coordination between academic and programmers, NGO and MoH in conducting research, not move beyond publication."
	"Most of the researches are done for requirement, and the researcher think when and how to finish. They did not think to make change on policy and action."
	"One of the big challenges is having Ministry of Health really buying in early to the research, so when the research is being planned, there's that level of buy-in so later the results can be

	translated. If MOH people are not involved early, it sometimes makes it much more challenging to translate”
	“Most of the time we have not experience to expand from pilot programs, learn from our lessons and to scale up to wider ones. Decision makers made decisions without questioning evidence and adequate information. We do not have such type of institutions which support evidence into policies there is some effort by professional institutions tried to influence.”
	“Program managers have no time to search evidences from various and wider documents. Leaders request evidence for influencing but we could not deliver evidence with short period of time. Better if the knowledge management and research advisors come on board at the ministry level.”
	“There is big gap and disconnection between methodological (public health) and technical (clinical) experts in research. Among clinicians there is methodological gap and time/workload to do research, no motivation and interest. Among public health experts, the did not know the real gap and problem because of limited subject matter knowledge thus they will continue of doing KAP studies… both do not want to come out from their comfort zone, there might be ego to ask support. The public health also does not push to work with clinicians. The whole system is organized to work separately.”
	“Our own guideline, policy and strategy are not designed using local evidence and knowledge. We usually use external evidence to make decisions. We undermine our own evidence.”
Importance of engaging community actors	“The community complains about the effect of research: ‘You always collect data but you did not make any change.’”
	“Most of the time there is a research dissemination plan to the community by researches while we reviewed for ethical clearance but not realized in actual terms.”
	“There is no community engagement except for data collection. In our context, being a research advisory committee is not practiced, [but] the community can also have priority problems, thus researchers can engage the community during research question development, geographic location, identifying the severity of problem”

	"On maternal health, from the women themselves, who can express the problem very well, and also the situation in their community. Religious leaders, for instance in Afar and Somali, they make decision in many aspects of RMCH… The main objective of the research is to solve the problem, showing and identifying the problem itself. When external body comes to us, they can act on the already identified problem. This help to direct towards community priority problem."
	"Currently we did community engagement in Jimma, we started to engage them from protocol development, it was an interesting lesson, we want to write that to share with others. The community had good input on the proposal."
	"Interpretation of the findings should be context specific – from program point of view, from policy environment etc. There are gaps in interpretation. Interpretation only has been viewed from researcher's perspective and lacks holistic approach."
Enablers of community-based research	"[Including the community in research] needs skill and it is a skill set: how to talk to the community, it needs protocol and guideline how engage community in research."
	"To involve, the community representative from the very beginning can bring sense of ownership of the problem."
	"There should be agreed research designed activities (involving stakeholders in research from the design phase) rather than inviting in the dissemination."
	"In newborn project was aimed to manage newborn sepsis through HEW for a mother denied referral to the health center was one of good example in which all of stakeholders (partners and all the health system actors- FMoH, regions, zones, woredas, PHCU including HEW) engaged in designing, work plan development, implementation, monitoring and evaluation. As a result, the findings easily got acceptance and research evidences used policy formulation, strategy setting and program planning."
	"When it is longitudinal, having community advisory board. For example, in abroad in the DSS sites there are community advisory aboard but here we do not have such practice. They contribute on identifying research questions, involve in data collection and on result

	dissemination. They are also part of it, it is an ideal scenario. If it is done like this for longitudinal studies, it is good and also possible to do so.”
	“The community should be part of the designing and result should be communicated to their level of understanding. Even the community can bring justification for the research findings. Using their own language, if we say to the community we found this from you, what do you think, and what are the reasons, it is good we have such practice. We intended so many times but we could not do that.”
Operational infrastructure improvements needed for MNCH research	“We do not have infrastructure. Usually people consider the hardware aspect of the infrastructure. But we do not have software programs, we are crackers. We do not have journal or subscribed database system. Our librarians are very traditional just they wait the one who is coming in and going out. There are changes on the internet capacity.”
	“Related to literature, we do have access only for free databases and open access journals. Sometimes, we request friends from abroad get article, if that needs subscription. We do not have database system to use clinical/patient data for research. In general, we do not have data management center to get support.”
	“Most of these institutions are pretty fragile. One reason is they often don't charge much overhead. Donor organizations hate overhead and feel it's an evil thing, money flushed down the drain, but overhead pays for the electricity and water, and most critically, internet access and access to medical journals for students… In countries like Ethiopia, students have a hell of a time getting access to articles, and they don’t know how to do literature searches, so that also affects uptake of the latest knowledge and latest interventions because decision-makers and researchers have trouble getting access to systematic reviews, to cutting edge journals that have paywalls, and students don’t get much teaching or guidance about how to do searches, a systematic review, what are different databases, what kind of literature you can access in each database.”
	“For research, the IT capacity is not so good. There is no platform like ODK, and other to collect data using tablets. But most of the time these things are accessed not from IT unit, it is of individual effort.”

	"Laboratory capacity in human resource and material is deficient – alarmingly bad to conduct a strong research or do landscape kind of research."
	"Even within health facility, a lot of research I've seen published is based on what's recorded in the clinical and medical records, and maybe not of the highest quality or most complete or accurate data."
	"Research financial utilization and management in universities another challenge for efferent use of resource. Being it is too tight and not convenient for researchers. The financial management system also creates frustration for researchers. Resource mobilization is not a challenge, but the current existed poor financial management in academic institutions created problems for smooth implementation. They might not able to provide financial reports even after six month of the implementation period. Grant management is a big deal and challenge than of resource solicitation/mobilization."
Funding priorities for MNCH research	"The current existed budget was not tailored to country health problem. It focused on the donors' interest, like WHO, UNICEF, not related to local health problem."
	"Few donors and NGOs conducting trial to identify effectiveness of some programs; however, they never work on root problem identification being they have they own interest as well as needs to adopt from global experience. There are no local efforts to conduct intervention research by identifying real RMNCH problem in the country. Most of the researches are donor driven and not well considered the local context. Most of the time it mainly relays on the global evidence (either form Asia or from Africa) than initiating from the local problem identification."

Appendix S2. Score distribution, average, and percentage agreement for all Delphi statements by topic

1. Improving the institutional MNCH research environment in Ethiopia

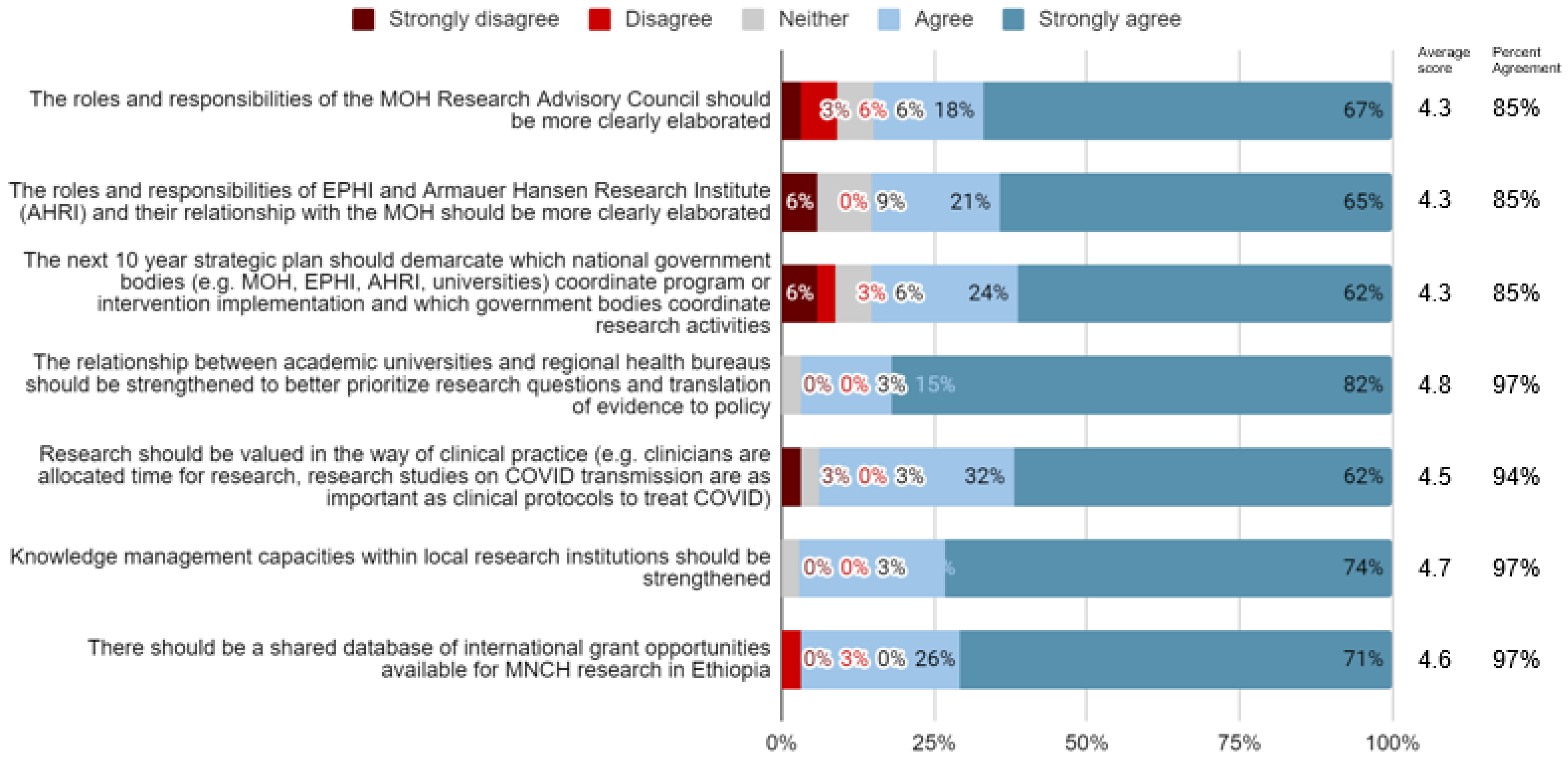

2. Improving MNCH research agenda-setting

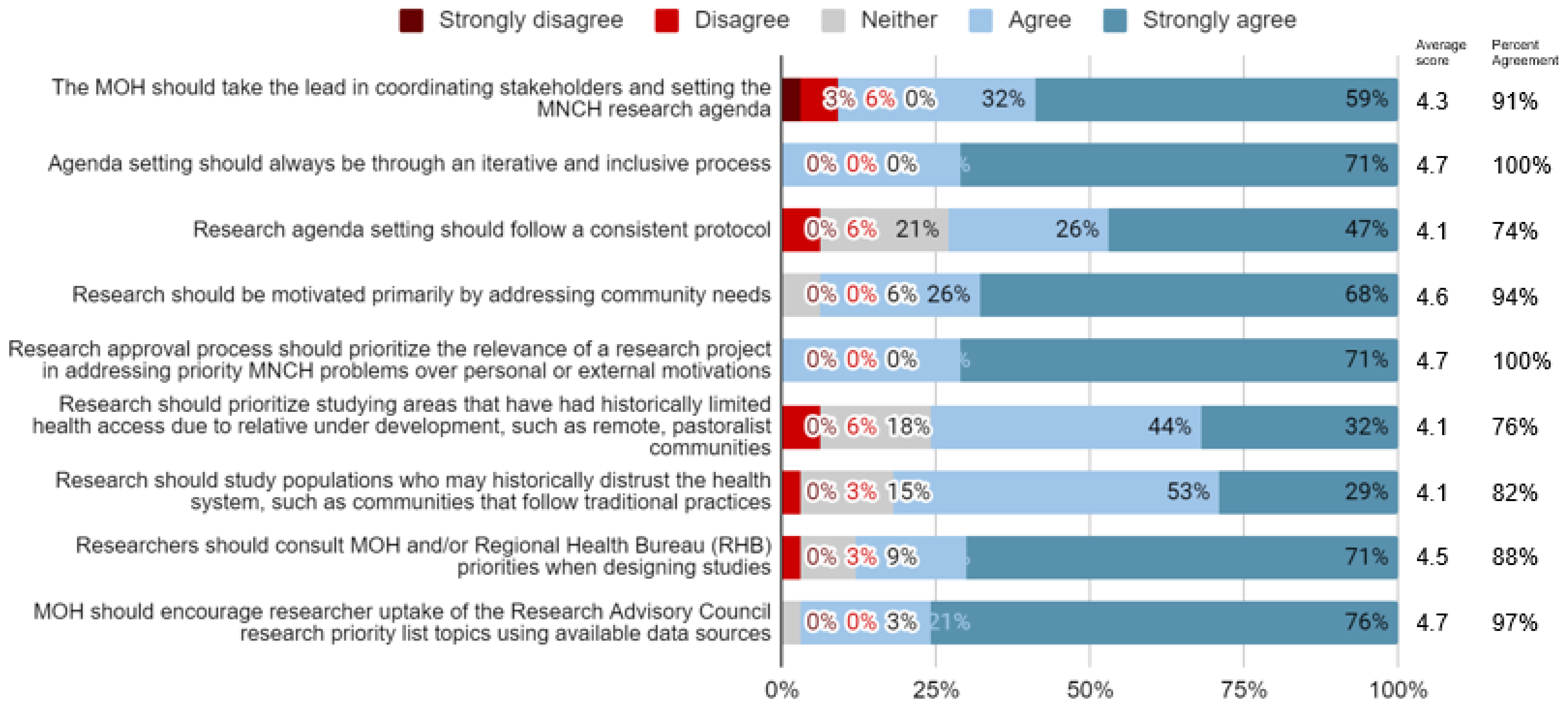

3. Research training priorities

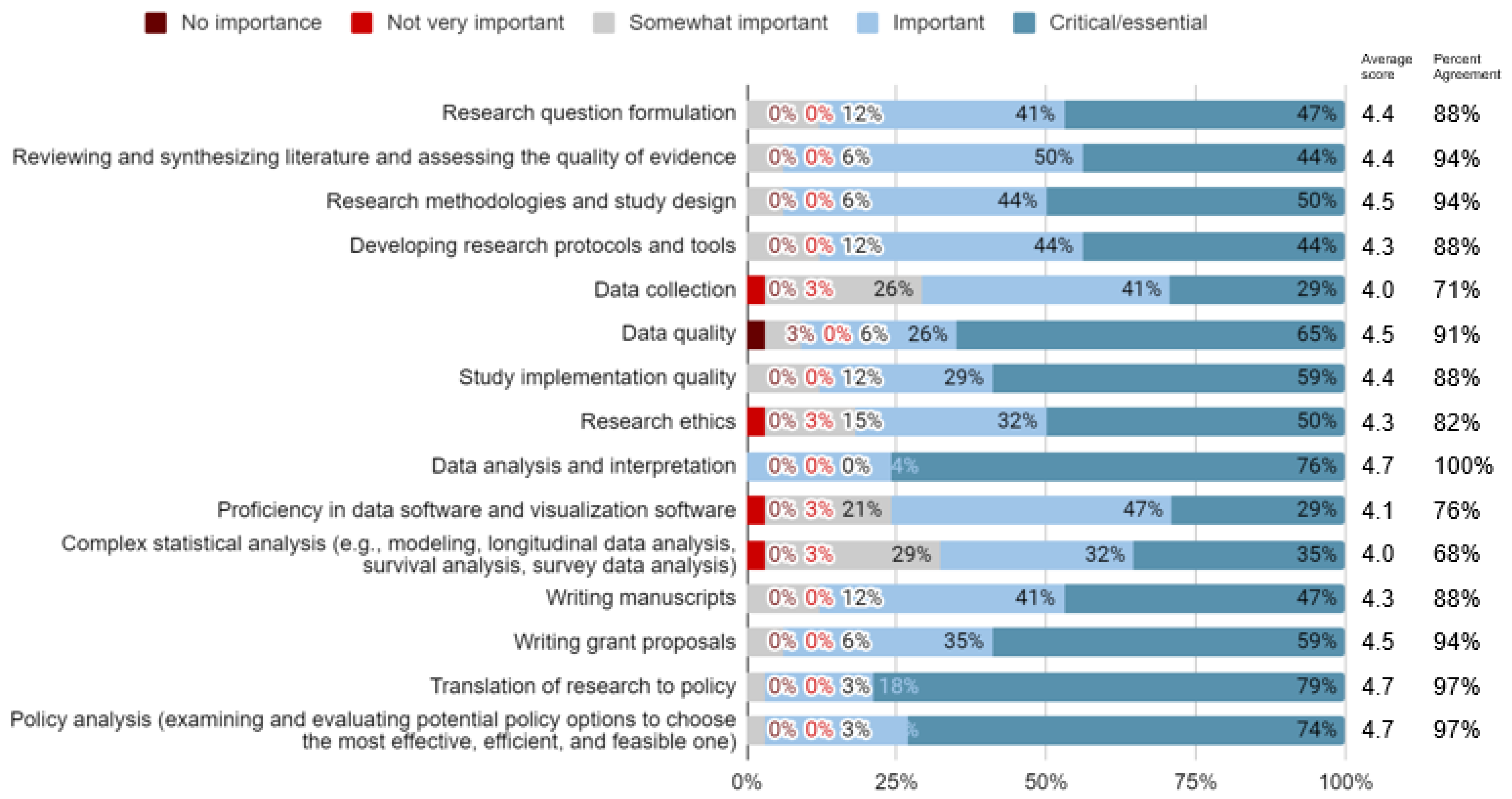

4. Improving academic research capacity and training in Ethiopia

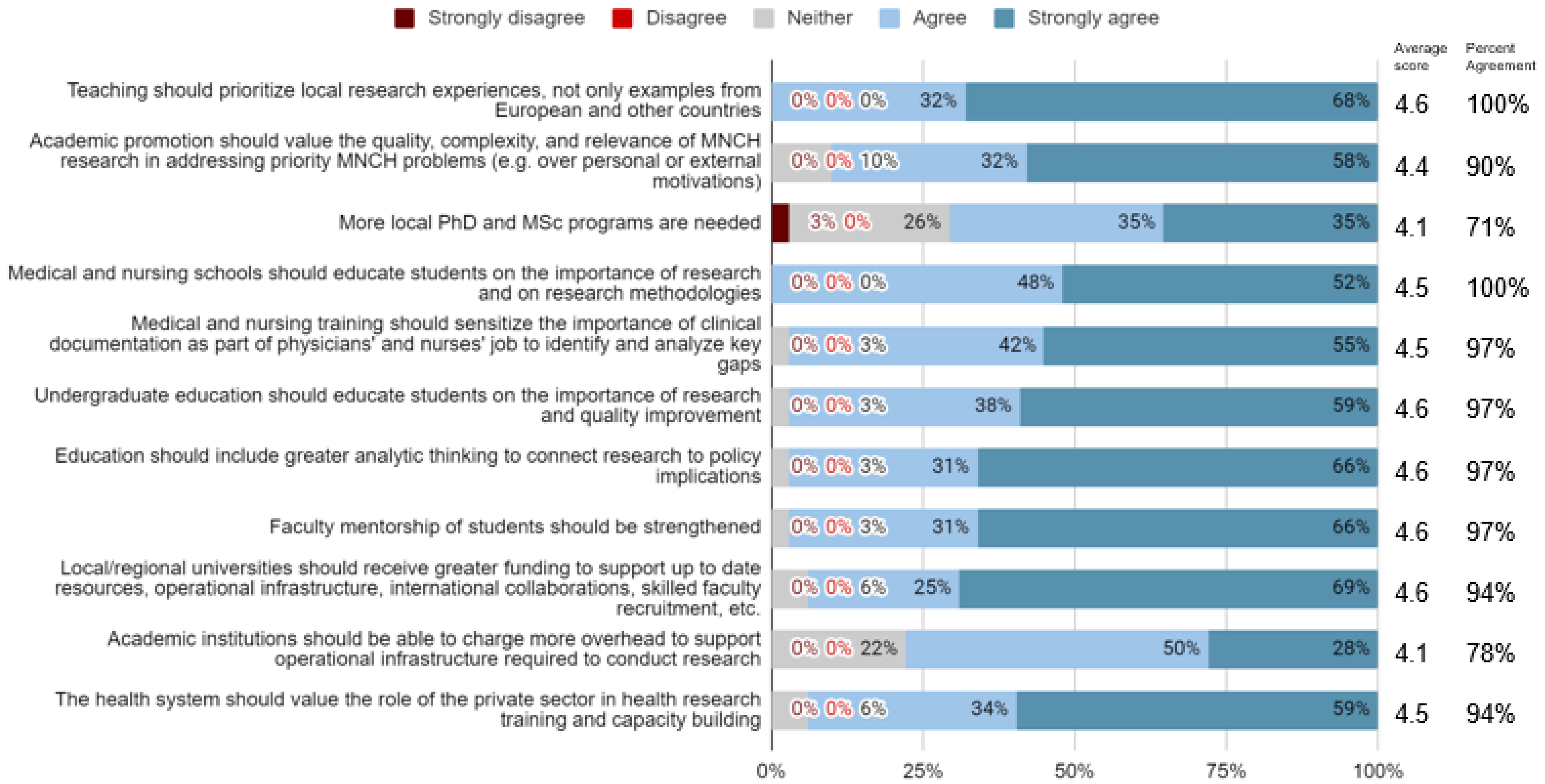

5. MNCH evidence sharing in Ethiopia

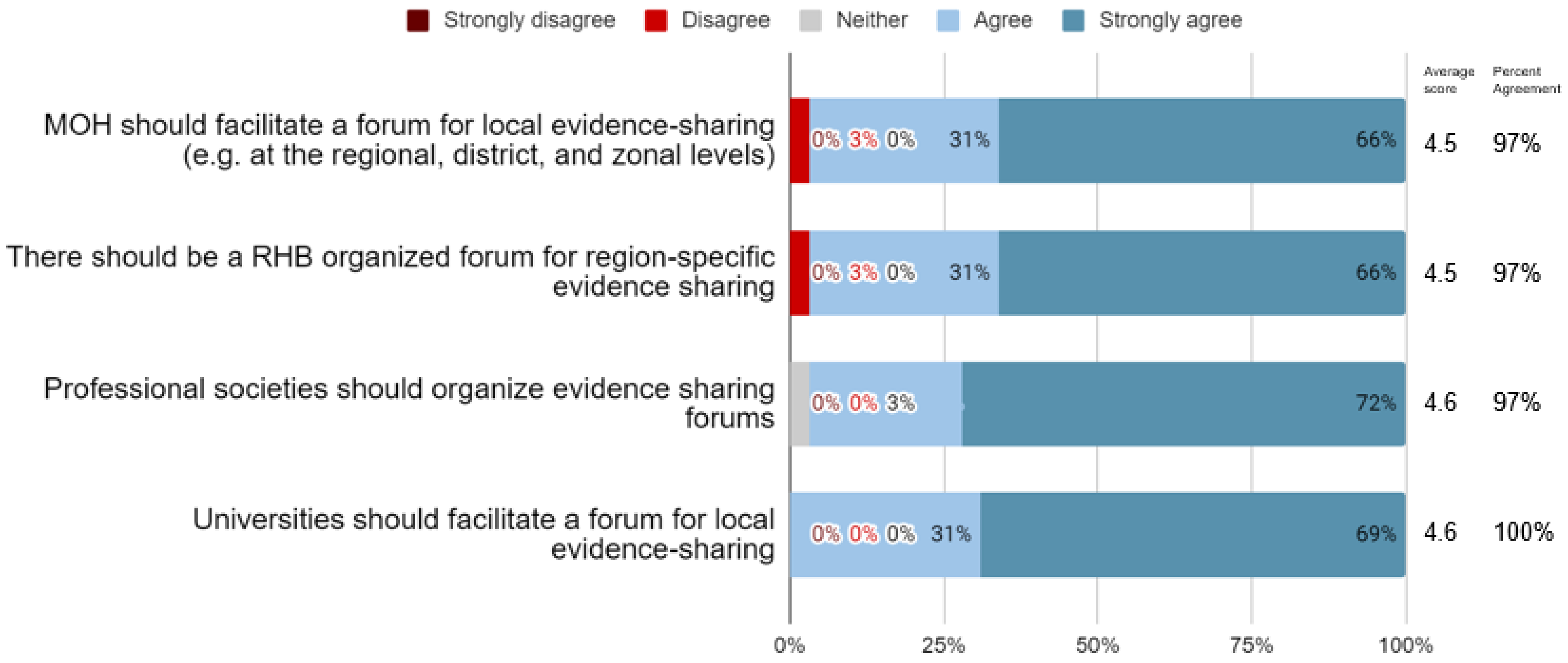

6. Improving research collaborations for MNCH in Ethiopia

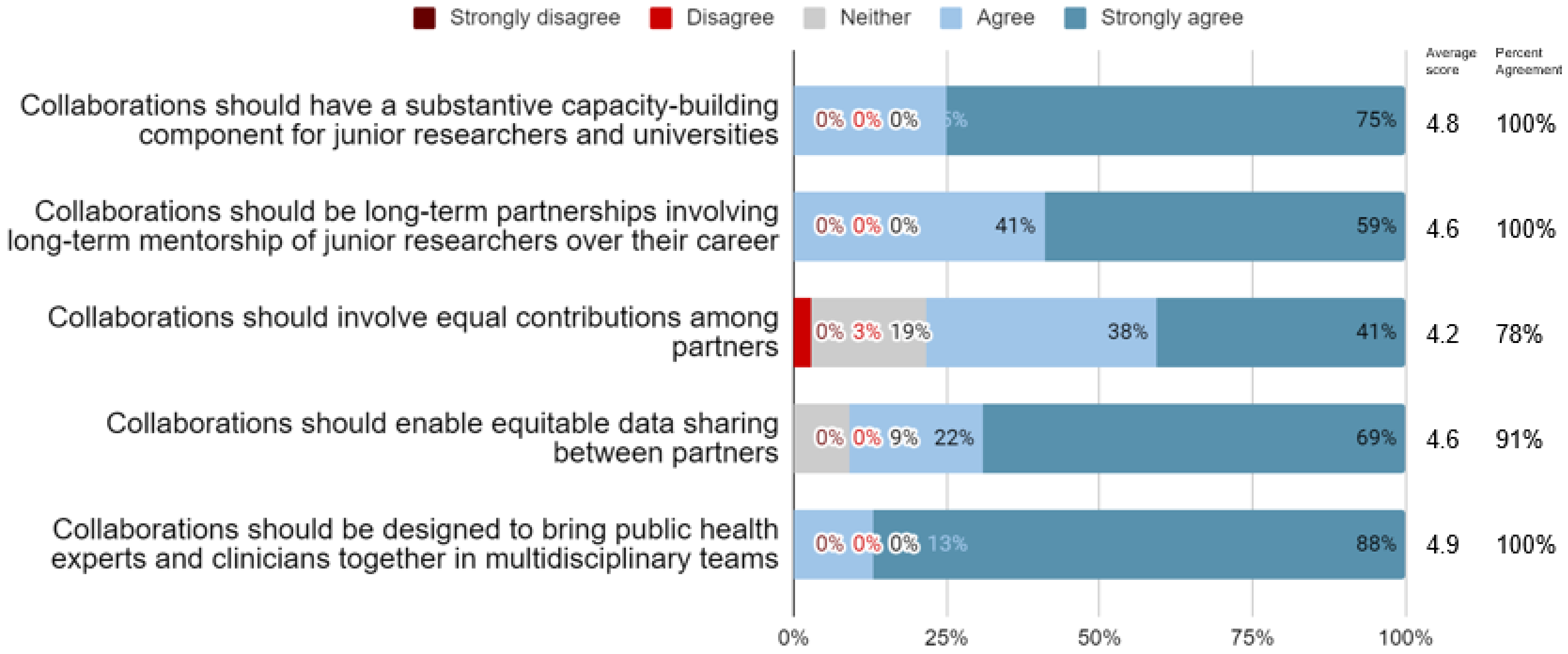

7. Metrics of success for an MNCH research network

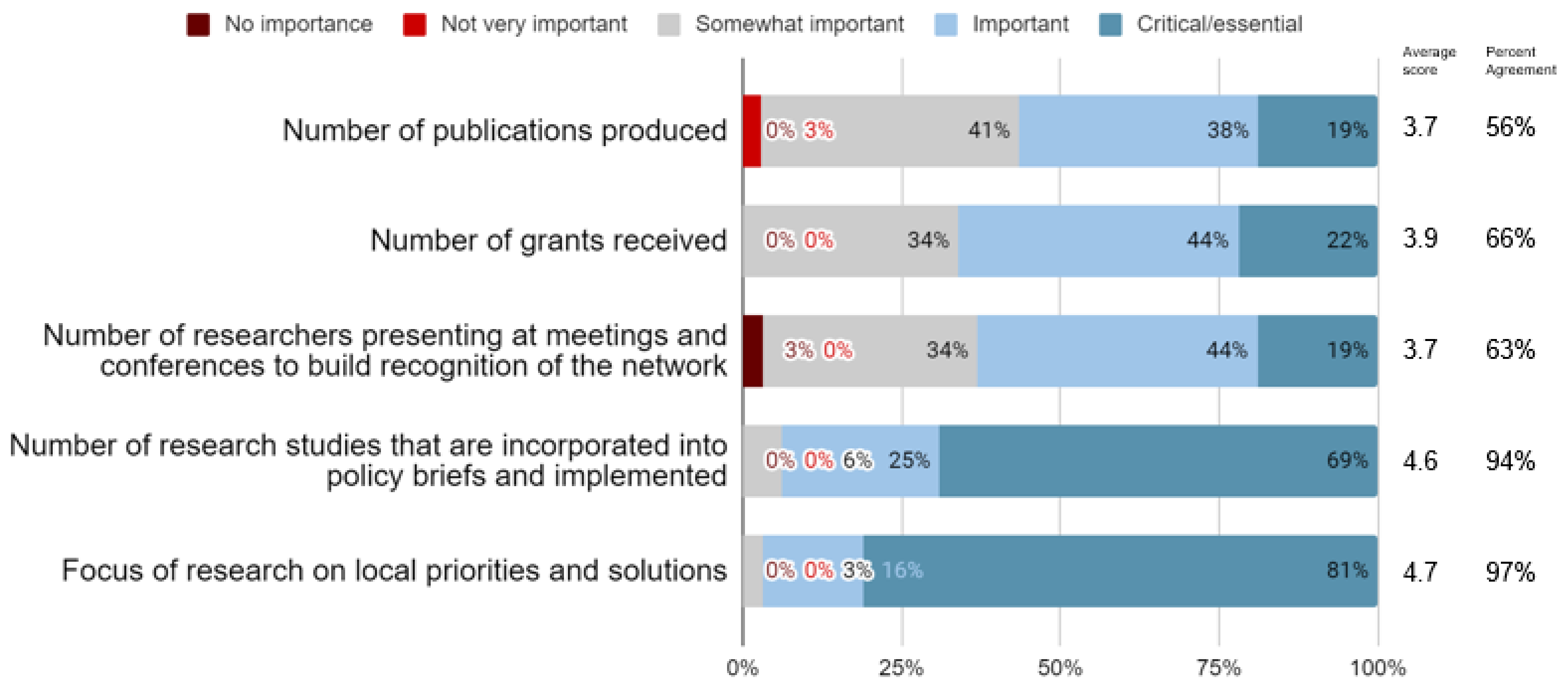

8. Enablers of the translation of MNCH research to program and policy action

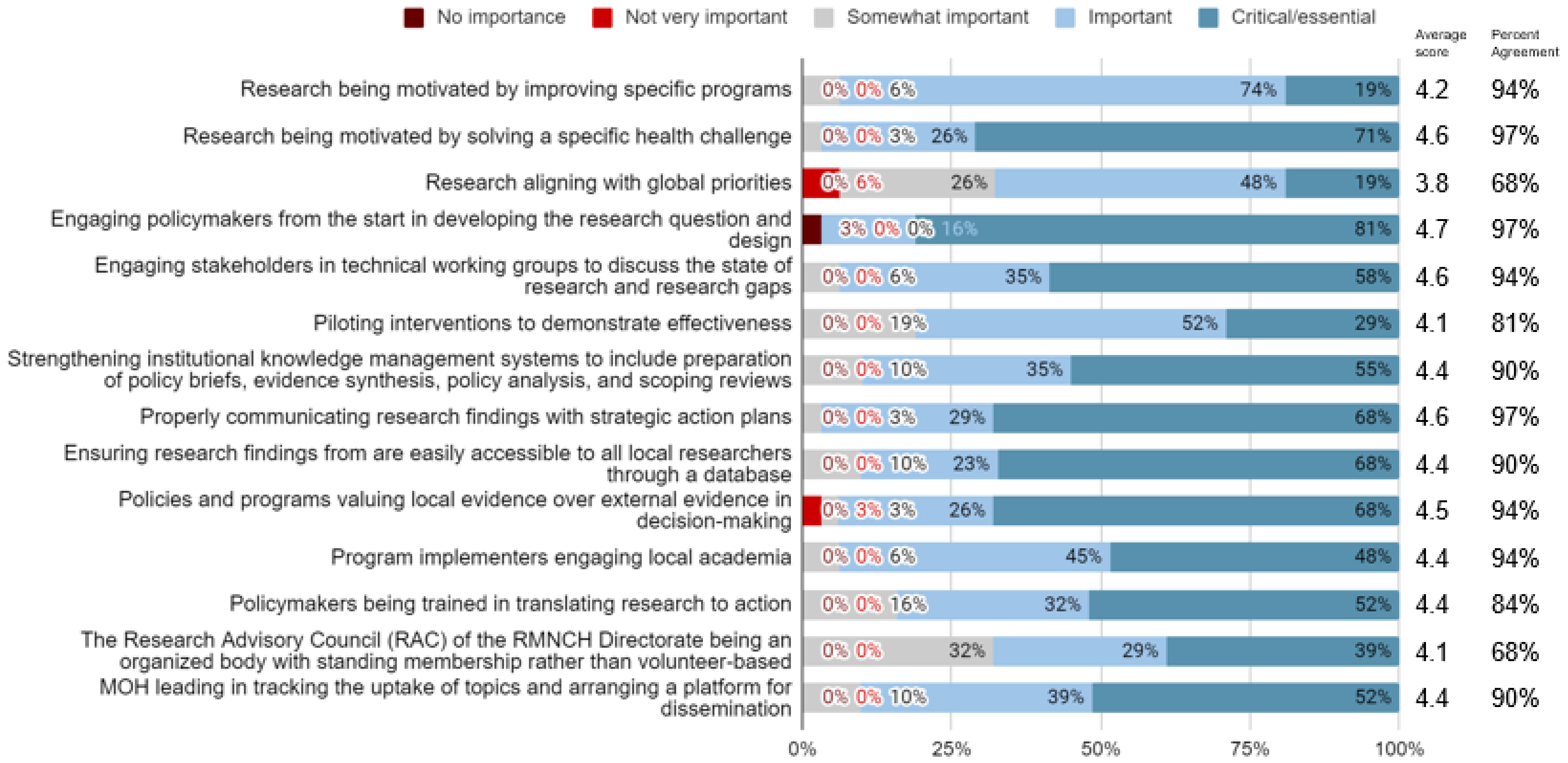

9. Importance of engaging community actors

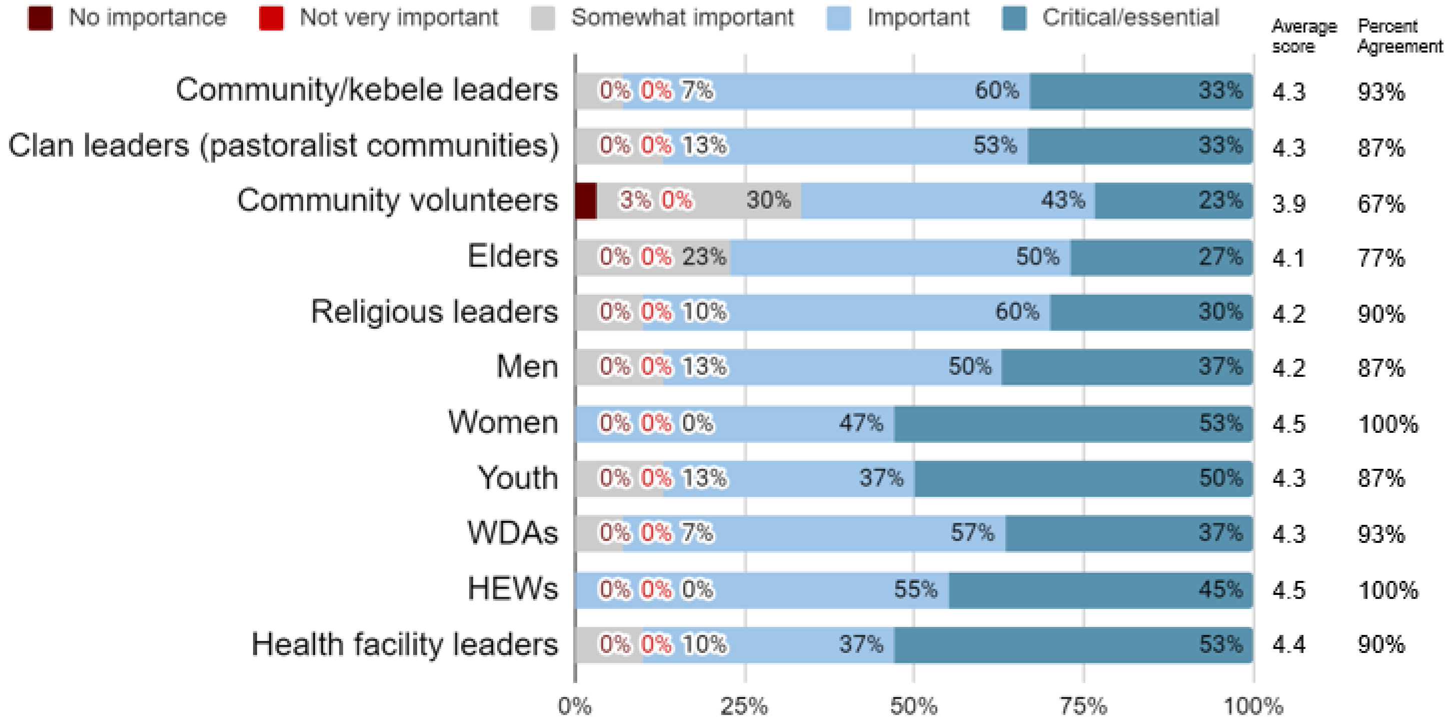

10. Enablers of community-based research

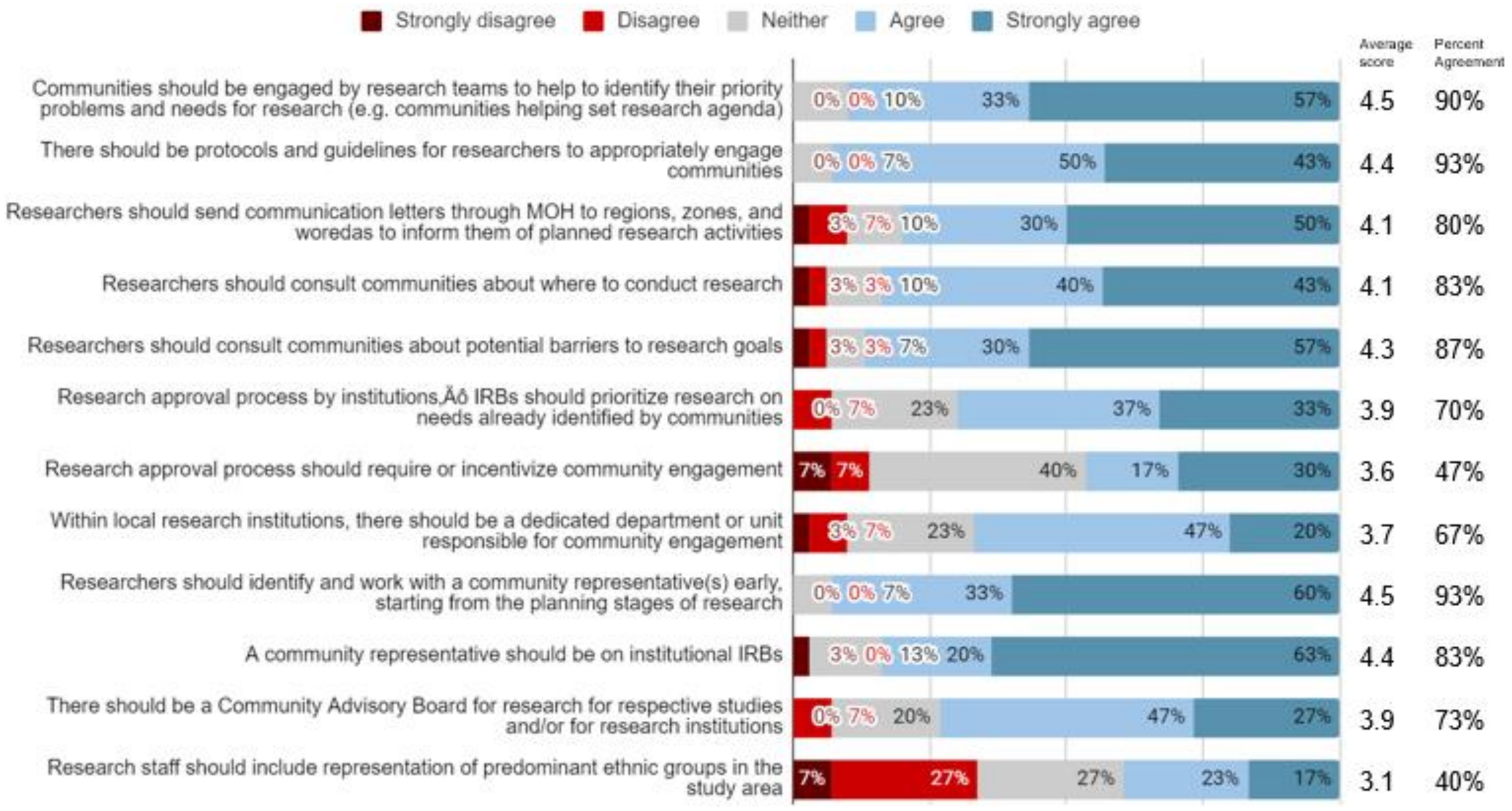

Strongly disagree
Disagree
Neither
Agree
Strongly agree
Average score
Percent Agreement
Communities should be engaged by research teams to help to identify their priority problems and needs for research (e.g. communities helping set research agenda)
0% 0% 10% 33% 57% 4.5 90%
There should be protocols and guidelines for researchers to appropriately engage communities
0% 0% 7% 50% 43% 4.4 93%
Researchers should send communication letters through MOH to regions, zones, and woredas to inform them of planned research activities
3% 7% 10% 30% 50% 4.1 80%
Researchers should consult communities about where to conduct research
3% 3% 10% 40% 43% 4.1 83%
Researchers should consult communities about potential barriers to research goals
3% 3% 7% 30% 57% 4.3 87%
Research approval process by institutions‚Äô IRBs should prioritize research on needs already identified by communities
0% 7% 23% 37% 33% 3.9 70%
Research approval process should require or incentivize community engagement
7% 7% 40% 17% 30% 3.6 47%
Within local research institutions, there should be a dedicated department or unit responsible for community engagement
3% 7% 23% 47% 20% 3.7 67%
Researchers should identify and work with a community representative(s) early, starting from the planning stages of research
0% 0% 7% 33% 60% 4.5 93%
A community representative should be on institutional IRBs
3% 0% 13% 20% 63% 4.4 83%
There should be a Community Advisory Board for research for respective studies and/or for research institutions
0% 7% 20% 47% 27% 3.9 73%
Research staff should include representation of predominant ethnic groups in the study area
7% 27% 27% 23% 17% 3.1 40%

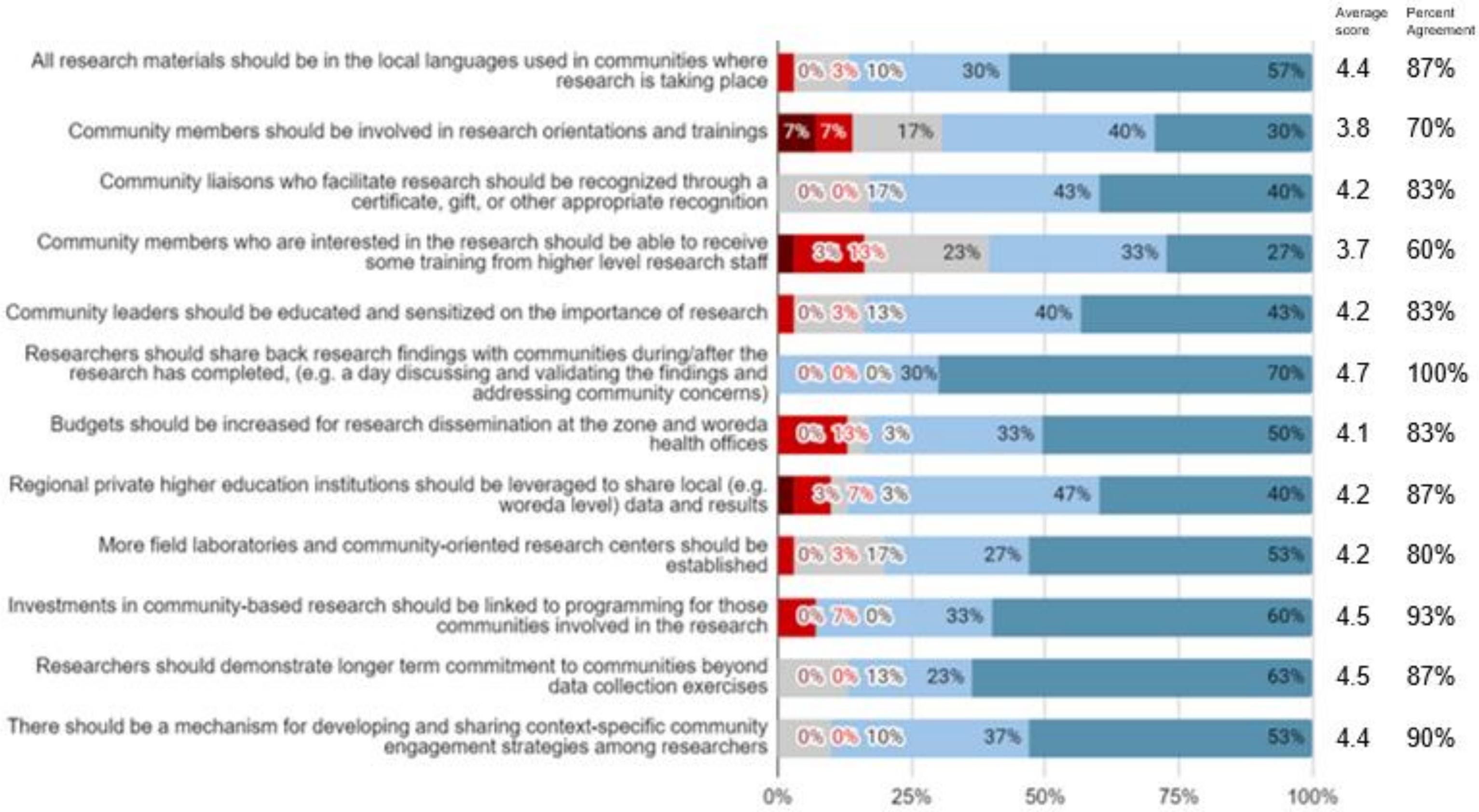

11. Operational infrastructure improvements needed for MNCH research

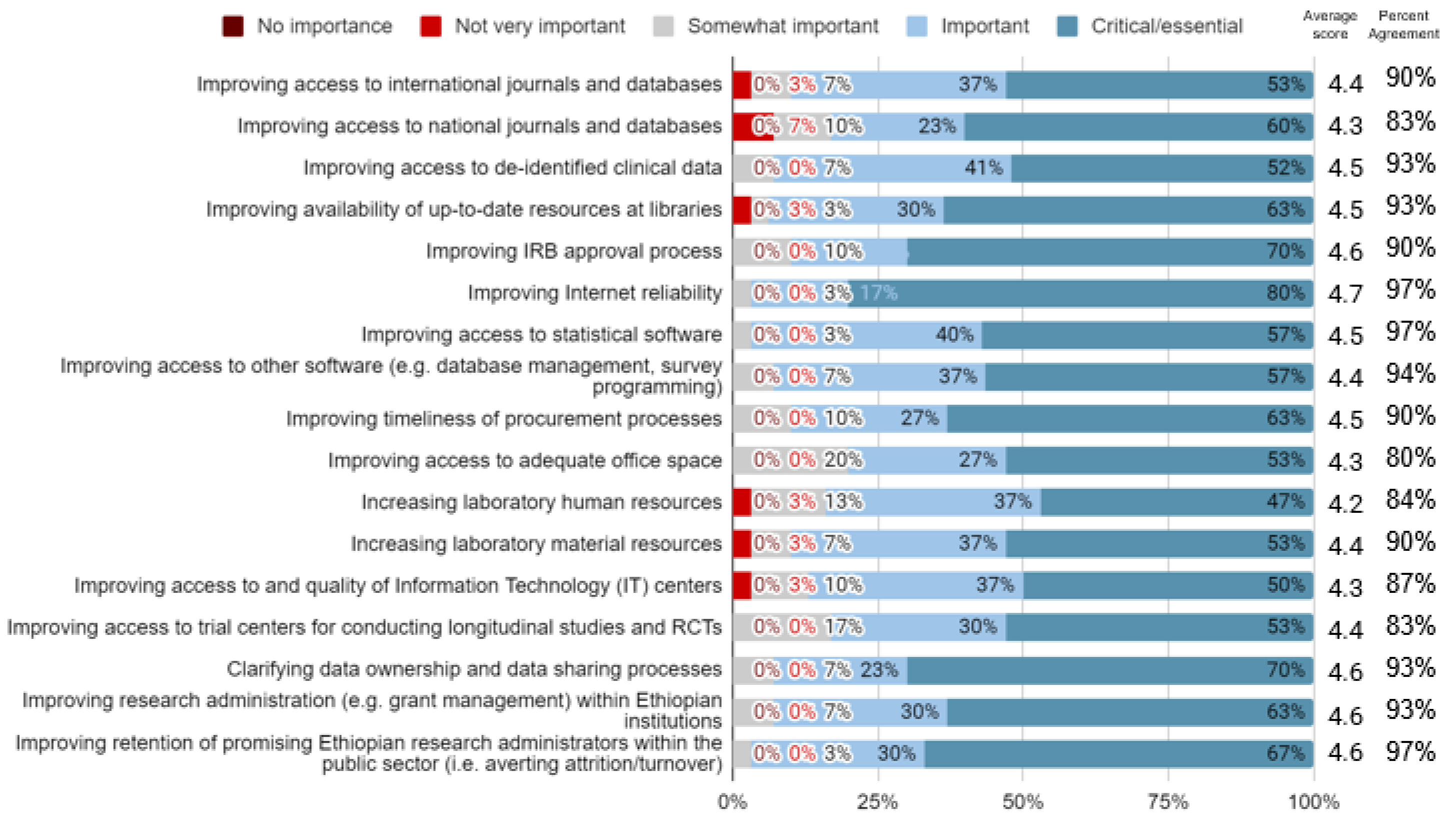

12. Funding priorities for MNCH research

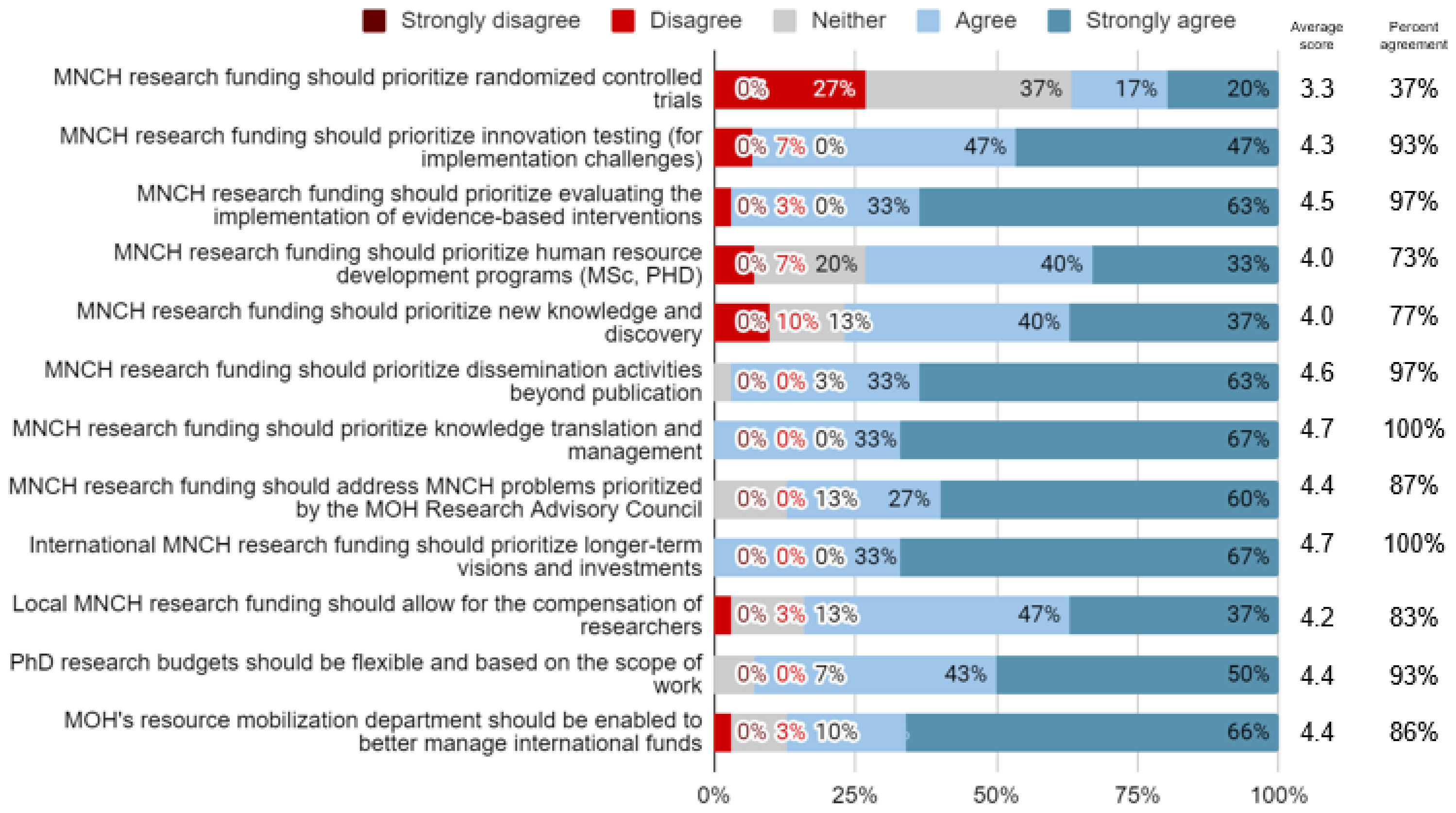
Strongly disagree
Disagree
Neither
Agree
Strongly agree
Average score
Percent agreement
MNCH research funding should prioritize randomized controlled trials
MNCH research funding should prioritize innovation testing (for implementation challenges)
MNCH research funding should prioritize evaluating the implementation of evidence-based interventions
MNCH research funding should prioritize human resource development programs (MSc, PHD)
MNCH research funding should prioritize new knowledge and discovery
MNCH research funding should prioritize dissemination activities beyond publication
MNCH research funding should prioritize knowledge translation and management
MNCH research funding should address MNCH problems prioritized by the MOH Research Advisory Council
International MNCH research funding should prioritize longer-term visions and investments
Local MNCH research funding should allow for the compensation of researchers
PhD research budgets should be flexible and based on the scope of work
MOH's resource mobilization department should be enabled to better manage international funds
0% 27% 37% 17% 20% 3.3 37%
0% 7% 0% 47% 47% 4.3 93%
0% 3% 0% 33% 63% 4.5 97%
0% 7% 20% 40% 33% 4.0 73%
0% 10% 13% 40% 37% 4.0 77%
0% 0% 3% 33% 63% 4.6 97%
0% 0% 0% 33% 67% 4.7 100%
0% 0% 13% 27% 60% 4.4 87%
0% 0% 0% 33% 67% 4.7 100%
0% 3% 13% 47% 37% 4.2 83%
0% 0% 7% 43% 50% 4.4 93%
0% 3% 10% 66% 4.4 86%
0%
25%
50%
75%
100%

Appendix S3. **Average score and percentage agreement for all Delphi statements, by percentage agreement**

Topic	Statement	Percent agreement	Average score
Improving MNCH research agenda-setting	Agenda setting should always be through an iterative and inclusive process	100%	4.7
Improving MNCH research agenda-setting	Research approval process should prioritize the relevance of a research project in addressing priority MNCH problems over personal or external motivations	100%	4.7
Research training priorities	Data analysis and interpretation	100%	4.7
Improving academic research capacity and training in Ethiopia	Teaching should prioritize local research experiences, not only examples from European and other countries	100%	4.6
Improving academic research capacity and training in Ethiopia	Medical and nursing schools should educate students on the importance of research and on research methodologies	100%	4.5
MNCH evidence sharing in Ethiopia	Universities should facilitate a forum for local evidence-sharing	100%	4.6
Improving research collaborations for MNCH in Ethiopia	Collaborations should be designed to bring public health experts and clinicians together in multidisciplinary teams	100%	4.9
Improving research collaborations for MNCH in Ethiopia	Collaborations should have a substantive capacity-building component for junior researchers and universities	100%	4.8
Improving research collaborations for MNCH in Ethiopia	Collaborations should be long-term partnerships involving long-term mentorship of junior researchers over their career	100%	4.6
Importance of engaging community actors	Women	100%	4.5
Importance of engaging community actors	HEWs	100%	4.5
Enablers of community-based research	Researchers should share back research findings with communities during/after the research has completed, (e.g. a day discussing and validating the findings and addressing community concerns)	100%	4.7
Priorities for MNCH research funding	MNCH research funding should prioritize knowledge translation and management	100%	4.7

Priorities for MNCH research funding	International MNCH research funding should prioritize longer-term visions and investments	100%	4.7
Improving the institutional MNCH research environment in Ethiopia	Knowledge management capacities within local research institutions should be strengthened	98%	4.7
Research training priorities	Policy analysis (examining and evaluating potential policy options to choose the most effective, efficient, and feasible one)	98%	4.7
Metrics of success for an MNCH research network	Focus of research on local priorities and solutions	97%	4.7
Enablers of the translation of MNCH research to program and policy action	Engaging policymakers from the start in developing the research question and design	97%	4.7
Operational infrastructure improvements for MNCH research	Improving Internet reliability	97%	4.7
Improving the institutional MNCH research environment in Ethiopia	The relationship between academic universities and regional health bureaus should be strengthened to better prioritize research questions and translation of evidence to policy	97%	4.8
Improving the institutional MNCH research environment in Ethiopia	There should be a shared database of international grant opportunities available for MNCH research in Ethiopia	97%	4.6
Improving MNCH research agenda-setting	MOH should encourage researcher uptake of the Research Advisory Council research priority list topics using available data sources	97%	4.7
Research training priorities	Translation of research to policy	97%	4.7
Improving academic research capacity and training in Ethiopia	Undergraduate education should educate students on the importance of research and quality improvement	97%	4.6
Improving academic research capacity and training in Ethiopia	Education should include greater analytic thinking to connect research to policy implications	97%	4.6
Improving academic research capacity and training in Ethiopia	Faculty mentorship of students should be strengthened	97%	4.6

Improving academic research capacity and training in Ethiopia	Medical and nursing training should sensitize the importance of clinical documentation as part of physicians‚Äô/nurses‚Äô job to identify and analyze key gaps	97%	4.5
MNCH evidence sharing in Ethiopia	Professional societies should organize evidence sharing forums	97%	4.6
MNCH evidence sharing in Ethiopia	MOH should facilitate a forum for local evidence-sharing (e.g. at the regional, district, and zonal levels)	97%	4.5
MNCH evidence sharing in Ethiopia	There should be a RHB organized forum for region-specific evidence sharing	97%	4.5
Enablers of the translation of MNCH research to program and policy action	Research being motivated by solving a specific health challenge	97%	4.6
Enablers of the translation of MNCH research to program and policy action	Properly communicating research findings with strategic action plans	97%	4.6
Operational infrastructure improvements for MNCH research	Improving retention of promising Ethiopian research administrators within the public sector (i.e. averting attrition/turnover)	97%	4.6
Operational infrastructure improvements for MNCH research	Improving access to statistical software	97%	4.5
Priorities for MNCH research funding	MNCH research funding should prioritize dissemination activities beyond publication	96%	4.6
Priorities for MNCH research funding	MNCH research funding should prioritize evaluating the implementation of evidence-based interventions	96%	4.5
Improving MNCH research agenda-setting	Research should be motivated primarily by addressing community needs	94%	4.6
Enablers of the translation of MNCH research to program and policy action	Policies and programs valuing local evidence over external evidence in decision-making	94%	4.5
Improving the institutional MNCH research environment in Ethiopia	Research should be valued in the way of clinical practice (e.g. clinicians are allocated time for research, research studies on COVID transmission are as important as clinical protocols to treat COVID)	94%	4.5
Research training priorities	Research methodologies and study design	94%	4.5

Research training priorities	Writing grant proposals	94%	4.5
Research training priorities	Reviewing and synthesizing literature and assessing the quality of evidence	94%	4.4
Improving academic research capacity and training in Ethiopia	Local/regional universities should receive greater funding to support up to date resources, operational infrastructure, international collaborations, skilled faculty recruitment, etc.	94%	4.6
Metrics of success for an MNCH research network	Number of research studies that are incorporated into policy briefs and implemented	94%	4.6
Importance of engaging community actors	WDAs	94%	4.3
Operational infrastructure improvements for MNCH research	Improving access to other software (e.g. database management, survey programming)	94%	4.4
Priorities for MNCH research funding	MNCH research funding should prioritize innovation testing (for implementation challenges)	94%	4.3
Improving academic research capacity and training in Ethiopia	The health system should value the role of the private sector in health research training and capacity building	93%	4.5
Enablers of the translation of MNCH research to program and policy action	Engaging stakeholders in technical working groups to discuss the state of research and research gaps	93%	4.6
Enablers of the translation of MNCH research to program and policy action	Program implementers engaging local academia	93%	4.4
Enablers of the translation of MNCH research to program and policy action	Research being motivated by improving specific programs	93%	4.2
Importance of engaging community actors	Community/kebele leaders	93%	4.3
Enablers of community-based research	Researchers should identify and work with a community representative(s) early, starting from the planning stages of research	93%	4.5
Enablers of community-based research	Investments in community-based research should be linked to programming for those communities involved in the research	93%	4.5
Enablers of community-based research	There should be protocols and guidelines for researchers to appropriately engage communities	93%	4.4

Operational infrastructure improvements for MNCH research	Clarifying data ownership and data sharing processes	93%	4.6
Operational infrastructure improvements for MNCH research	Improving research administration (e.g. grant management) within Ethiopian institutions	93%	4.6
Operational infrastructure improvements for MNCH research	Improving access to de-identified clinical data	93%	4.5
Operational infrastructure improvements for MNCH research	Improving availability of up-to-date resources at libraries	93%	4.5
Priorities for MNCH research funding	PhD research budgets should be flexible and based on the scope of work	93%	4.4
Research training priorities	Data quality	91%	4.5
Enablers of the translation of MNCH research to program and policy action	Ensuring research findings from are easily accessible to all local researchers through a database	91%	4.4
Enablers of the translation of MNCH research to program and policy action	MOH leading in tracking the uptake of topics and arranging a platform for dissemination	91%	4.4
Improving MNCH research agenda-setting	The MOH should take the lead in coordinating stakeholders and setting the MNCH research agenda	91%	4.3
Improving research collaborations for MNCH in Ethiopia	Collaborations should enable equitable data sharing between partners	91%	4.6
Enablers of the translation of MNCH research to program and policy action	Strengthening institutional knowledge management systems to include preparation of policy briefs, evidence synthesis, policy analysis, and scoping reviews	90%	4.4
Importance of engaging community actors	Health facility leaders	90%	4.4
Enablers of community-based research	There should be a mechanism for developing and sharing context-specific community engagement strategies among researchers	90%	4.4

Operational infrastructure improvements for MNCH research	Improving timeliness of procurement processes	90%	4.5
Operational infrastructure improvements for MNCH research	Improving access to international journals and databases	90%	4.4
Operational infrastructure improvements for MNCH research	Increasing laboratory material resources	90%	4.4
Improving academic research capacity and training in Ethiopia	Academic promotion should value the quality, complexity, and relevance of MNCH research in addressing priority MNCH problems (e.g. over personal or external motivations)	90%	4.4
Importance of engaging community actors	Religious leaders	90%	4.2
Enablers of community-based research	Communities should be engaged by research teams to help to identify their priority problems and needs for research (e.g. communities helping set research agenda)	90%	4.5
Operational infrastructure improvements for MNCH research	Improving IRB approval process	90%	4.6
Improving MNCH research agenda-setting	Researchers should consult MOH and/or Regional Health Bureau (RHB) priorities when designing studies	89%	4.5
Research training priorities	Developing research protocols and tools	88%	4.3
Research training priorities	Research question formulation	88%	4.4
Research training priorities	Study implementation quality	88%	4.4
Research training priorities	Writing manuscripts	88%	4.3
Importance of engaging community actors	Youth	87%	4.3
Importance of engaging community actors	Men	87%	4.2
Enablers of community-based research	Regional private higher education institutions should be leveraged to share local (e.g. woreda level) data and results	87%	4.2

Operational infrastructure improvements for MNCH research	Improving access to and quality of Information Technology (IT) centers	87%	4.3
Priorities for MNCH research funding	MNCH research funding should address MNCH problems prioritized by the MOH Research Advisory Council	87%	4.4
Priorities for MNCH research funding	MOH's resource mobilization department should be enabled to better manage international funds	87%	4.4
Enablers of community-based research	All research materials should be in the local languages used in communities where research is taking place	87%	4.4
Enablers of community-based research	Researchers should consult communities about potential barriers to research goals	87%	4.3
Importance of engaging community actors	Clan leaders (pastoralist communities)	86%	4.3
Improving the institutional MNCH research environment in Ethiopia	The roles and responsibilities of EPHI and Armauer Hansen Research Institute (AHRI) and their relationship with the MOH should be more clearly elaborated	86%	4.3
Improving the institutional MNCH research environment in Ethiopia	The next 10 year strategic plan should demarcate which national government bodies (e.g. MOH, EPHI, AHRI, universities) coordinate program or intervention implementation and which government bodies coordinate research activities	86%	4.3
Enablers of community-based research	Researchers should demonstrate longer term commitment to communities beyond data collection exercises	86%	4.5
Improving the institutional MNCH research environment in Ethiopia	The roles and responsibilities of the MOH Research Advisory Council should be more clearly elaborated	85%	4.3
Enablers of the translation of MNCH research to program and policy action	Policymakers being trained in translating research to action	84%	4.4
Operational infrastructure improvements for MNCH research	Increasing laboratory human resources	84%	4.2
Priorities for MNCH research funding	Local MNCH research funding should allow for the compensation of researchers	84%	4.2
Enablers of community-based research	A community representative should be on institutional IRBs	83%	4.4
Enablers of community-based research	Community liaisons who facilitate research should be recognized through a certificate, gift, or other appropriate recognition	83%	4.2

Enablers of community-based research	Community leaders should be educated and sensitized on the importance of research	83%	4.2
Enablers of community-based research	Researchers should consult communities about where to conduct research	83%	4.1
Enablers of community-based research	Budgets should be increased for research dissemination at the zone and woreda health offices	83%	4.1
Operational infrastructure improvements for MNCH research	Improving access to trial centers for conducting longitudinal studies and RCTs	83%	4.4
Operational infrastructure improvements for MNCH research	Improving access to national journals and databases	83%	4.3
Improving MNCH research agenda-setting	Research should study populations who may historically distrust the health system, such as communities that follow traditional practices	82%	4.1
Research training priorities	Research ethics	82%	4.3
Enablers of the translation of MNCH research to program and policy action	Piloting interventions to demonstrate effectiveness	81%	4.1
Enablers of community-based research	More field laboratories and community-oriented research centers should be established	80%	4.2
Enablers of community-based research	Researchers should send communication letters through MOH to regions, zones, and woredas to inform them of planned research activities	80%	4.1
Operational infrastructure improvements for MNCH research	Improving access to adequate office space	80%	4.3
Improving research collaborations for MNCH in Ethiopia	Collaborations should involve equal contributions among partners	79%	4.2
Improving academic research capacity and training in Ethiopia	Academic institutions should be able to charge more overhead to support operational infrastructure required to conduct research	78%	4.1
Importance of engaging community actors	Elders	77%	4.1
Priorities for MNCH research funding	MNCH research funding should prioritize new knowledge and discovery	77%	4

Improving MNCH research agenda-setting	Research should prioritize studying areas that have had historically limited health access due to relative under development, such as remote, pastoralist communities	76%	4.1
Research training priorities	Proficiency in data software and visualization software	76%	4.1
Enablers of community-based research	There should be a Community Advisory Board for research for respective studies and/or for research institutions	74%	3.9
Improving MNCH research agenda-setting	Research agenda setting should follow a consistent protocol	73%	4.1
Priorities for MNCH research funding	MNCH research funding should prioritize human resource development programs (MSc, PHD)	73%	4
Research training priorities	Data collection	70%	4
Improving academic research capacity and training in Ethiopia	More local PhD and MSc programs are needed	70%	4.1
Enablers of community-based research	Research approval process by institutions‚Äô IRBs should prioritize research on needs already identified by communities	70%	3.9
Enablers of community-based research	Community members should be involved in research orientations and trainings	70%	3.8
Enablers of the translation of MNCH research to program and policy action	The Research Advisory Council (RAC) of the RMNCH Directorate being an organized body with standing membership rather than volunteer-based	68%	4.1
Research training priorities	Complex statistical analysis (e.g., modeling, longitudinal data analysis, survival analysis, survey data analysis)	67%	4
Enablers of the translation of MNCH research to program and policy action	Research aligning with global priorities	67%	3.8
Enablers of community-based research	Within local research institutions, there should be a dedicated department or unit responsible for community engagement	67%	3.7
Metrics of success for an MNCH research network	Number of grants received	66%	3.9
Importance of engaging community actors	Community volunteers	66%	3.9
Metrics of success for an MNCH research network	Number of researchers presenting at meetings and conferences to build recognition of the network	63%	3.7

Enablers of community-based research	Community members who are interested in the research should be able to receive some training from higher level research staff	60%	3.7
Metrics of success for an MNCH research network	Number of publications produced	57%	3.7
Enablers of community-based research	Research approval process should require or incentivize community engagement	47%	3.6
Enablers of community-based research	Research staff should include representation of predominant ethnic groups in the study area	40%	3.1
Priorities for MNCH research funding	MNCH research funding should prioritize randomized controlled trials	37%	3.3

Percentage agreement is the percent of responses that scored the statement 4 or 5 out of 5 (either "agree" or "strongly agree")
